# Microwave-Assisted Synthesis of Some Potential Bioactive Imidazolium-Based Room-Temperature Ionic Liquids

**DOI:** 10.3390/molecules23071727

**Published:** 2018-07-15

**Authors:** Ahmed H. Albalawi, Wael S. El-Sayed, Ateyatallah Aljuhani, Saud M. Almutairi, Nadjet Rezki, Mohamed R. Aouad, Mouslim Messali

**Affiliations:** 1 Department of Chemistry, Taibah University, Al-Madina Al-Mounawara 30002, Saudi Arabia; a-albalawi@hotmail.com (A.H.A.); ateyatallah@hotmail.com (A.A.); nadjetrezki@yahoo.fr (N.R.); aouadmohamedreda@yahoo.fr (M.R.A.); 2 Department of Biology Department, Taibah University, Al-Madina Al-Mounawara 30002, Saudi Arabia; waelsme@yahoo.com; 3 Microbiology Department, Faculty of Science, Ain Shams University, Cairo 11566, Egypt; 4 King Abdulaziz City for Science and Technology, Riyadh 11442, P.O. Box 6086, Saudi Arabia; sm_ksa_9@hotmail.com; 5 Laboratoire de Chimie & Electrochimie des Complexes Métalliques (LCECM) USTO-MB, University of Sciences and Technology Mohamed Boudiaf, BP 1505 Oran, El M nouar, Algeria

**Keywords:** ionic liquids, imidazolium, green synthesis, microwave irradiation, toxicity, antibacterial activity

## Abstract

An environmentally-friendly and easy synthesis of a series of novel functionalized imidazolium-based ionic liquids (ILs) is described under both the conventional procedure and microwave irradiation. The structures of newly synthesized room-temperature ionic liquids (RTILs) were established by different spectral analyses. All ILs (**1**–**14**) were screened for their in vitro antimicrobial activity against a panel of clinically isolated bacteria. The results of the minimum inhibitory concentration (MIC) and the minimum bactericidal concentration (MBC) showed that some of the tested ILs are very promising anti-bacterial agents especially those containing an alkyl chain with a phenyl group (most notably **1**, **2**, **12**, and **13**).

## 1. Introduction

A few decades ago, room-temperature ionic liquids (RTILs and salts usually composed of large organic cations and inorganic or organic anions) appeared as an emerging class of ecofriendly compounds alternative to volatile organic compounds (VOCs) due to their outstanding physical and chemical properties such as negligible vapor pressure, excellent thermal and chemical stability, outstanding dissolving capacity, excellent ionic conductivity, non-flammability, and recyclability [1,2,3].

The above-mentioned characteristics make them strongly attractive for applications in a myriad of different fields and have, therefore, been investigated for a broad range of applications including as potential corrosion inhibitors [4,5], in stereo-selective polymerization [6], as liquid crystals [7], in separation technology [8], in electrochemistry [9,10,11], in pharmacology [12,13], and catalysis [14,15,16].

However, possession of these properties does not necessarily make them a greener alternative. Therefore, several works on the biodegradability and toxicity of some ionic liquids (ILs) have recently been published with the aim of understanding the relationship between the molecular structure of ILs and their micro-toxicity [17,18,19,20,21,22,23,24]. Furthermore, bacteria are a satisfactory starting point for examining IL toxicity since they have short generation times compared with other living organisms [25]. This has indirectly led to the realization that some ILs exhibit very interesting antimicrobial characteristics against a wide panel of important microorganisms [24,25,26,27,28,29,30].

The preparation of these salts by conventional procedures requires a great deal of time and energy (72 h under reflux) [31,32]. After the first use by Varma [33], microwave (MW)-assisted reactions in the synthesis of ILs as a green alternative process have received greater attention [34,35]. The most common benefits of MW irradiation are: (1) very rapid reactions (frequently a few minutes) brought about by high and uniform temperatures combined with pressure effects, (2) higher degree of purity achieved due to short residence time at high temperatures, and (3) yields are often better, obtained within shorter times, and have purer products [36].

Therefore, the main goal of the present work was the synthesis of a new family of room-temperature functionalized imidazolium-based ionic liquids. The series was selected based on diverse structural features (including n- alkyl chains, ester, ether, alcohol, or cyano groups). Additionally, the newly synthesized water-soluble RTILs were investigated for their anti-microbial activity in order to understand the effect of the presence of such functional groups in these new ionic liquids on their toxicity.

## 2. Results and Discussion

### 2.1. Chemistry

**Scheme 1:**
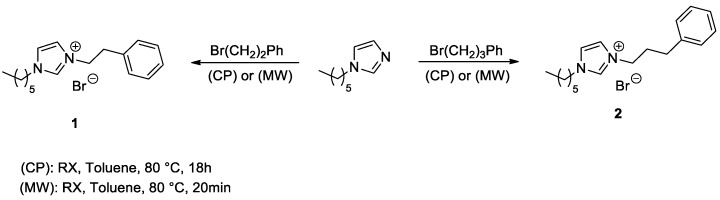
Synthesis of novel ionic liquids (ILs) with alkyl chain containing an ester group **1** and **2**.

Our recently-published results motivated us to continue the investigations [37,38] in order to prepare a new series of potential bioactive functionalized ILs. In this paper, we describe the synthesis of a new family of room-temperature ionic liquids (RTILs) **1**–**14** using a conventional procedure under microwave-assisted reaction conditions (Scheme 1).

The first hexylimidazolium-based derivatives **1** and **2** were prepared with the treatment of 1-hexylimidazole with (2-bromoethyl) benzene and (3-bromopropyl) benzene, respectively, in toluene at 80 °C for 18 h (Scheme 1).

The quaternization reaction took place through the nucleophilic attack of the Sp^2^-nitrogen imidazole atom, which acts as a nucleophile in the nucleophilic displacement of halogen on the alkyl halide to afford the corresponding imidazolium bromides **1** and **2** in 71–72% yield as viscous liquids after washing with dry ethyl acetate and solvent removal (Table 1).

The microwave-assisted synthesis of the first hexylimidazolium-based derivatives **1** and **2** was carried out in the CEM Discover monomode system with a strict control of power (300 W), a temperature of 80 °C, and high stirring during the reaction process. This process was implemented in order to shorten the reaction time and to increase the yields obtained under conventional methods. The formation of the IL was monitored as the reaction progressed from a clear homogenous solution to two phases because ILs are insoluble in toluene. The effect of the microwave was investigated and the results indicated that satisfactory yields were obtained in short reaction times compared with the previously mentioned conventional preparation (Table 1).

1-Hexyl-3-(3-phenylpropyl)-1*H*-imidazol-3-ium bromide (**2**) was selected as a model IL to discuss the obtained spectroscopic data used to confirm the formation of the imidazolium-based ILs **1** and **2**.

The *N*-alkylation of 1-hexylimidazole with (3-bromopropyl)benzene afforded 1-hexyl-3-(3-phenylpropyl)-1*H*-imidazol-3-ium bromide (**2**), which resulted in the appearance of a triplet estimated at around 0.83 ppm due to the methyl protons of the hexyl group. Since IL **2** contains eight methylene groups, three methylene protons appeared as a multiplet at 1.24 ppm and two methylene protons appeared as quintet, respectively, at 2.15 ppm and 2.61 ppm. The three other methylene protons appeared, respectively, as triplets at 2.61 ppm for CH_2_*CH*_2_Ph and at 4.20 ppm and 4.26 ppm for N*CH*_2_CH_2_. The H-NMR spectrum for **2** also contained two doublet signals at 7.91 ppm and 7.93 ppm and one singlet signal at 9.51 ppm, which were attributed to the imidazolium protons. The phenyl protons of the pendent aromatic rings in IL **2** were observed at 7.20 ppm to 7.27 ppm.

The C-NMR spectrum of IL **2** contained signals of the eight CH_2_, which appeared, respectively, at 49.1 ppm and 49.3 ppm for (NCH_2_) and the six other methylene groups were observed at their usual chemical shifts, which include 22.4, 25.6, 29.8, 31.0, 31.4, and 32.2 ppm. The C-NMR spectrum also displayed the signal of the CH_3_ group, which appeared at 14.3 ppm. Furthermore, the C-NMR spectrum contained signals corresponding to aromatic carbons including those of the imidazolium ring in the range of δ_C_ 122.9 ppm to 141.0 ppm. The obtained results were confirmed without ambiguity by the ^13^C-DEPT-NMR.

The success of this new strategy for the *N*-alkylation of 1-hexylimidazole was also confirmed by the Fourier transform infrared (FT-IR) spectra of ILs **2** and **3**. The FT-IR spectrum of IL **2** contained an absorption band around 2980 cm^−1^, which indicated the presence of (C=CH). Furthermore, it contained a peak at 2910 cm^−1^, which was consistent with the presence of CH_2_ and CH_3_ groups. Lastly, the mass spectrum of IL **2** contained peaks consistent with the desired mass ions [M − Br]^+^ known as 271.330 *m*/*z*.

After optimization of the reaction conditions, we continued to synthesize novel room-temperature ionic liquids. In this paper, we report the synthesis of a variety of new functionalized imidazolium-based ionic liquids tethered alkyl chain with ester functionality.

**Scheme 2:**
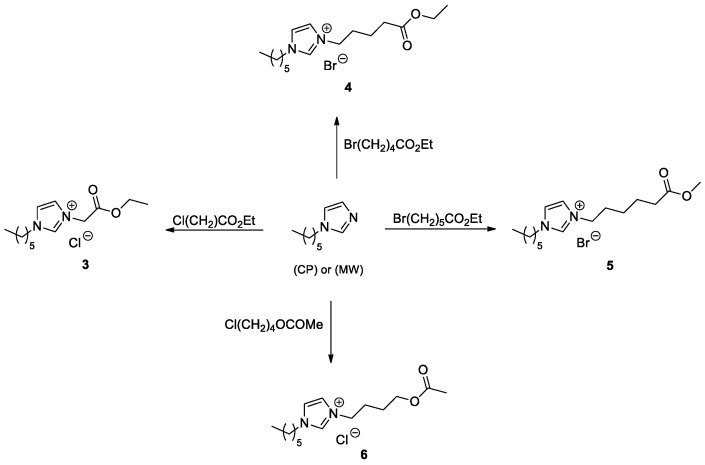
Synthesis of novel ILs **3**–**6** with an alkyl chain containing an ester group.

The quaternization reaction took place through the nucleophilic attack of lone pair nitrogen imidazole atom on alkyl halides containing an ester group to afford the corresponding imidazolium halides **3**–**6** in 69–79% yield as viscous liquids after washing with dry ethyl acetate and solvent removal (Scheme 2).

Microwave-assisted reactions were deployed for the synthesis of ILs **3**–**6** with the same goal. The previously mentioned advantages of microwave-assisted synthesis were observed by the use of this eco-friendly technology, the large reduction of reaction time, the simplicity in handling and processing, and the increase of the reaction yield (Table 2).

The structures of the halogenated imidazolium-based ionic liquids **3**–**6** were established on the basis of their spectral data (^1^H-NMR, ^13^C-NMR, FT-IR, mass, and elemental analysis). From this novel series ILs **3**–**6**, 3-(2-ethoxy-2-oxoethyl)-1-hexyl-1*H*-imidazol-3-ium chloride (**3**) was selected as a model IL to discuss the obtained spectroscopic data used to confirm its formation.

Since there are some parts of IL **3** that were present in the already discussed IL **2**, we insisted on new peaks. From the H-NMR spectrum of IL **3**, the characteristic singlet at δ_H_ 5.36 for two proton units was assigned to the N*CH*_2_CO and the characteristic ethyl ester protons appeared as a triplet and quartet at 1.22 ppm and 4.21 ppm, respectively, which confirmed the success of the quaternization step.

Furthermore, the C-NMR spectrum of IL **3** contained a signal consistent with the presence of a carbonyl group (CO) at δ_C_ 167.4 ppm. The signals of the CH_2_ and CH_3_ of the ester group (OCH_2_CH_3_) appeared, respectively, at 64.2 ppm and 14.3 ppm. The peak for (NCH_2_CO) appeared at around 50 ppm, which was confirmed clearly by APT-C-NMR. The five other methylene groups were observed at their usual chemical shifts. The structure of IL **3** was also confirmed by the FT-IR spectrum in which an absorption band at 1745 cm^−1^ clearly indicated the presence of a carbonyl group (C=O) belonging to an ester. Lastly, the mass spectrum of IL **3** contained a peak consistent with the desired mass ions [M − Cl]^+^ known as 239.172 *m*/*z*.

**Scheme 3:**
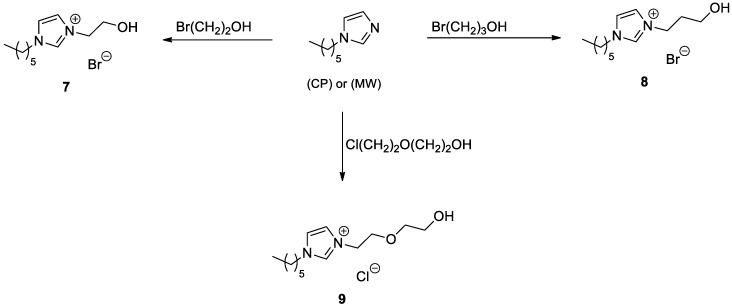
Synthesis of novel ILs **7**–**9** with alkyl chain containing an alcohol group.

In the same way, the synthesis of a new series of room-temperature ILs based on an imidazolium moiety and a tethered alkyl chain with alcohol functionality was investigated under both conventional and microwave-assisted procedures (Scheme 3).

The previously reported advantages of using microwave irradiation were observed (Table 3). The success of the alkylation was confirmed based on the spectral data of the resulted ILs **7**–**9** (IR, ^1^H-NMR, ^13^C-NMR, mass spectroscopy, and elemental analysis). The ionic liquid 1-hexyl-3-(2-hydroxyethyl)-1*H*-imidazol-3-ium bromide (**7**) was taken as a model compound to discuss the obtained spectroscopic data used to confirm the quaternization reaction of the 1-hexylimidazole by 2-bromoethanol.

Structural elucidation of ionic liquid **7** was deduced from its H-NMR spectrum, which confirmed the incorporation of an OH group. Therefore, a broad signal for (CH_2_CH_2_O*H*) was observed at δ_H_ 4.84 ppm and two triplets for (*CH_2_CH_2_*OH) appeared respectively at δ_H_ 3.81 and 4.28 ppm. The formation of IL **7** was also confirmed by C-NMR and the APT-^13^C-NMR analysis, which clearly showed the presence of methylene carbons at δ_C_ 52.1 ppm and 59.9 ppm belonging to the (CH_2_CH_2_OH) group. The FT-IR spectrum of IL **7** contained an absorption band at around 3350 cm^−1^, which indicated the presence of a hydroxyl group belonging to an alcohol. In addition, the structure of compound **7** was also confirmed by the electron impact mass spectrum, which showed a molecular ion peak [M − Br]^+^ at 197.064 *m*/*z*.

**Scheme 4:**
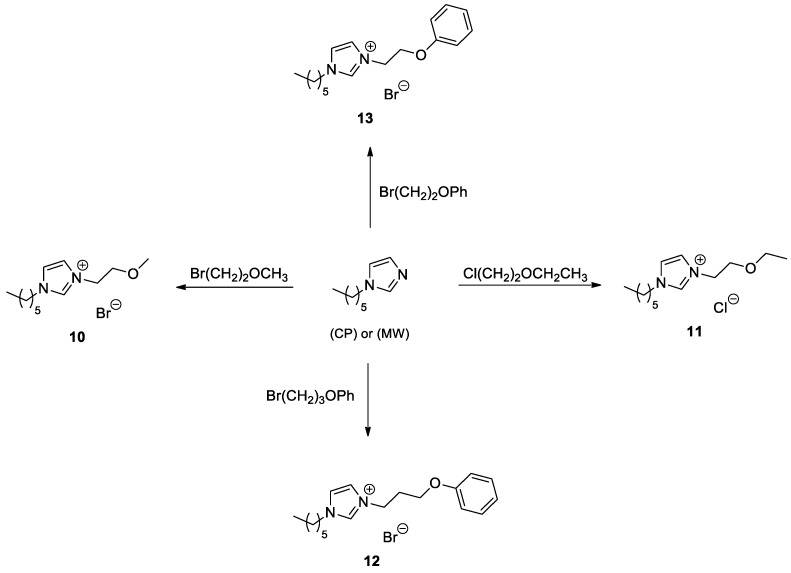
Synthesis of novel ILs **10**–**13** with an alkyl chain containing an ether group.

The synthesis of another series of new functionalized imidazolium-based ionic liquids derivatives was carried out under the same conditions. This time, the focus was on imidazolium-based ionic liquids containing an alkyl chain with ether functionality (Scheme 4).

The previously reported advantages of using microwave irradiation were observed, which include the short time of reaction and improved yield (Table 4).

Structures of the desired ionic liquids **10**–**13** were confirmed by their ^1^H-NMR, ^13^C-NMR, FT-IR, and mass spectra. The ionic liquid 1-hexyl-3-(3-phenoxypropyl)-1*H*-imidazol-3-ium bromide (**13**) was taken as a model compound to discuss the obtained spectroscopic data used to confirm the quaternization reaction of 1-hexylimidazole by (3-bromopropoxy) benzene.

The structure of IL **13** was first confirmed by H-NMR analysis, which resulted in the appearance of two methylene protons as triplets at 4.45 ppm for N*CH*_2_CH_2_OPh and at 3.98 ppm for CH_2_*CH*_2_OPh_._ The phenyl protons of the pendent aromatic rings in IL **13** were observed in the range of 6.84 ppm to 7.20 ppm. The C-NMR spectrum of ILs **13** contained signals CH_2_, which appeared respectively at 65.9 ppm for (CH_2_CH_2_O) and at 46.9 for (NCH_2_CH_2_O). Furthermore, the C-NMR spectrum contained signals corresponding to aromatic carbons including those of the imidazolium ring in the range of δ_C_ 114.8 ppm to 158.5 ppm. The obtained results were confirmed without ambiguity by APT-^13^C-NMR. The structure of IL **13** was also confirmed by the FT-IR spectrum in which an absorption band around 1250 cm^−1^ clearly indicated the presence of a (C-O) belonging to an ether group. Furthermore, the mass spectrum of IL **13** displayed molecular ion peak [M − Br]^+^ at 286.806 *m*/*z*.

**Scheme 5:**
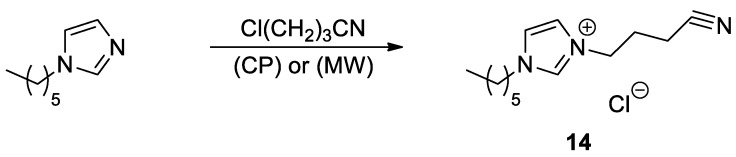
Synthesis of novel IL **14** with an alkyl chain containing a cyano group.

Finally, the synthesis of the last ionic liquid in this first part of the present work known as 3-(3-cyanopropyl)-1-hexyl-1*H*-imidazol-3-ium chloride (**14**) (Scheme 5) was also carried out under both conventional procedure and microwave irradiation. Its structure was confirmed by its ^1^H-NMR, ^13^C-NMR, FT-IR, and mass spectra.

From the H-NMR spectrum of IL **14**, two triplets for N*CH*_2_CH_2_*CH*_2_CN appeared, respectively, at δ_H_ 2.65 and 4.33 ppm. The C-NMR spectrum of IL **14** contained signals consistent with the presence of a quaternary carbon of a cyano group (CN) at δ_C_ 120.1 ppm. The peak for (CH_2_CN) appeared at δ_C_ 14.0 ppm. All other methylene groups were observed at their usual chemical shifts, which were confirmed clearly by APT-^13^C-NMR. However, the FT-IR clearly indicated a peak at around 2125 cm^−1^, which was consistent with the presence of a CN group. The mass spectral data revealed the presence of a molecular ion peak [M − Cl]^+^ at 220.112 *m*/*z*, which is shown by the formation of compound **14**.

### 2.2. Antibacterial Properties

#### 2.2.1. Inhibition Zone (IZ)

Several reports have recently been published showing that ILs possess promising biological activities [39,40]. Encouraged by these results, we proceeded to evaluate the antimicrobial activities of ILs **1**–**14** against seven strains of bacteria including four Gram-positive strains (i.e., *Staphylococcus aureus*, *Bacillus cereus*, *Bacillus amyloliquefaciens*, and *Escherichia coli*) and three Gram-negative strains (i.e., *Acinetobacter baumannii*, *Klebsiella pneumonia*, and *Pseudomonas aeruginosa*)*.*

The bacterial strains used in this study were selected to cover a wide range of potential bacterial pathogens with serious clinical manifestation. They have been recommended as reference strains by the European Committee on Antimicrobial Susceptibility Testing (EUCAST) for antimicrobial susceptibility testing [41]. These bacterial strains are known for their frequent development of antimicrobial drug resistance and, therefore, require continuous monitoring. *E. coli*, *P. aeruginosa*, and *S. aureus* are most commonly used in clinical studies [42]*. K. pneumoniae* and *A. baumani* are known to cause nosocomial infections and acquire simultaneous resistance mechanisms [43,44]. *Bacillus* spp. have also been included in this study as Gram-positive strains, which are known as a serious causative agent for food poisoning. Due to their potential pathogenic properties, drugs possessing antibacterial activity against these strains could be regarded as convenient and promising antibacterial agents.

The antibacterial activities of ILs **1**–**14** were tested in vitro against the selected bacteria mentioned above and were determined by using the agar diffusion method with Mueller-Hinton agar medium.

The results of the IZ experiments are displayed in Table 5 and showed that all newly synthesized ILs except **3** and **14** exhibited different levels of antibacterial activity towards the seven tested strains compared with known strong standard drugs including ampicillin, rifampicin, clindamycin, and kanamycin.

We deduced that ILs **1**, **2**, **8**, **12**, and **13** were very potent against *S. aureus*, *B. cereus*, and *B. amyloliquefaciens* with inhibition zone (IZ) values ranging within 32 mm to 40 mm, 16 mm to 27 mm, and 23 mm to 57 mm, respectively. They exhibited the highest activity towards these tested microorganisms in the current study at a concentration of 100 µg/mL.

In addition to other pathogens, ILs **1**, **2**, **9**, **12**, and **13** were also strongly effective against *A. baumanni*, *B. cereus*, and *P. aeruginosa* with inhibition zone (IZ) values ranging within 12–17 mm, 32–40 mm, and 13–23 mm, respectively. It is important to mention that all tested standard drugs are not active against *P. aeruginosa.* Yet, only ILs **12** and **13** exhibited an excellent degree of antibacterial activity regarding the inhibition zone experiments even when comparing their results with those of the tested standard drugs.

#### 2.2.2. Minimum Inhibitory Concentration (MIC)

ILs **1**–**14** were selected in order to test their in vitro minimum inhibitory concentration (MIC) and minimum bactericidal concentration (MBC). The obtained results are summarized in Table 6.

Specifically, 1-hexyl-3-(2-phenoxyethyl)-1*H*-imidazol-3-ium bromide (**12**) showed broad-spectrum antibacterial activity and was very effective against all tested pathogenic bacteria. IL **13** exhibited an MIC value of 4 µg/mL for both *B. amyloliquefaciens* and *S. aureus*. It also showed an MIC value of 8 µg/mL for *B. cereus*, *K. pneumoniae*, and *P. aeruginosa* and 16 µg/mL for *E. coli* and *A. baumannii*. 1-Hexyl-3-(3-phenoxypropyl)-1*H*-imidazol-3-ium bromide (**13**) was also very promising with estimated MIC values of 2 µg/mL and 4 µg/mL for *B. amyloliquefaciens* and *S. aureus*, respectively. It also showed MIC values of 8 µg/mL, 16 µg/mL, 32 µg/mL, and 64 µg/mL, respectively, for *B. cereus*, *K. pneumoniae*, *E. coli*, and *A. baumannii.* Both compounds contain alkyl chains with a phenoxy group.

The results of MIC experiments displayed in Table 6 indicate the excellent results exhibited by ILs **1**, **2**, **12**, and **13**, which showed excellent activity against most of the tested bacterial strains with an MIC range of 2 µg/mL to 16 µg/mL, which is equal to or less than those recorded for ampicillin, rifampicin, clindamycin, and kanamycin.

Next to ILs **12** and **13** come ILs **1** and **2**, which were the second most efficient compounds with broad-spectrum antibacterial activity. 1-Hexyl-3-(3-phenylpropyl)-1*H*-imidazol-3-ium chloride (**2**) exhibited MIC values of 4 µg/mL and 8 µg/mL for both *B. amyloliquefaciens* and *S. aureus*, respectively*.* It showed an MIC value of 16 µg/mL for both *B. cereus* and *K. pneumoniae*. Its exhibited MIC value was 32 µg/mL for both *E. coli* and *P. aeruginosa* and it had a moderate activity against *A. baumannii* with an estimated MIC value of 64 µg/mL. However, 1-hexyl-3-phenethyl-1*H*-imidazol-3-ium bromide (**1**) exhibited MIC values of 4 µg/mL for both *B. amyloliquefaciens* and *S. aureus*. It showed an MIC value of 32 µg/mL for *K. pneumoniae* and had moderate activity against *B. cereus* and *E. coli* with an estimated MIC value of 64 µg/mL. Like ILs **12** and **13**, ILs **1** and **2** contain an alkyl chain with a phenyl group in their structures.

Furthermore, 1-hexyl-3-(2-(2-hydroxyethoxy)ethyl)-1*H*-imidazol-3-ium chloride (**9**) was effective only against *K. pneumoniae* with an estimated MIC value of 16 µg/mL. It showed a moderate activity against *S. aureus* and *E. coli* with an estimated MIC value of 16 µg/mL. Lastly, ILs **5**–**7**, **10**, and **11** showed moderate-to-weak antibacterial activities only against *S. aureus* and *B. amyloliquefaciens* with estimated MIC values ranging from 64 µg/mL to 128 µg/mL.

## 3. Conclusions

In the present work, the synthesis of 14 novel room-temperature ionic liquids (RTILs) was successfully carried out through the quaternization of 1-hexylimidazole by treating with different functionalized alkyl halides using an efficient eco-friendly microwave-assisted procedure in comparison with the conventional procedure. We found that the simple, convenient, and efficient microwave-assisted reaction could significantly enhance the synthetic yield of these RTILs, which decreased the reaction time. The structures of the novel ILs were established based on their H-NMR, C-NMR, FT-IR, and mass spectra.

However, due to their broad spectrum and potent antibacterial activity, these newly synthesized ILs (**1**, **2**, **12**, and **13** in particular) represent very promising antibacterial agents. These results confirm our recently published results and let us attribute this without ambiguity to the presence of an alkyl chain with a phenyl group. Other ILs did not show high antimicrobial toxicity.

## 4. Materials and Methods

### 4.1. Experimental

Commercially available solvents and reagents were purified according to standard procedures. All new compounds were synthesized and characterized by ^1^H-NMR, ^13^C-NMR, FT-IR, mass and elemental analysis. The NMR spectra were measured in DMSO-*d*_6_ or CDCl_3_ at room temperature. Chemical shifts (δ) were reported in ppm on a scale calibrated for tetramethylsilane (TMS), which is used as an internal standard (Bruker, Fällanden, Switzerland). The mass spectra were measured with a Bruker Maldi TOF MS (Bruker). IR spectra were recorded in NaCl disc on a Schimadzu 8201 PC, FT-IR spectrophotometer (υ_max_ in cm^−1^) (Shimadzu Scientific Instruments INC, Canby, OR, USA). The elemental analyses were given by using the 2400 Series II CHNS/O Elemental Analyzer (Perkin Elmer, Waltham, MA, USA). The microwave-assisted reactions were performed using a controllable single-mode microwave reactor (CEM Corp., Matthews, NC, USA), CEM Discovery, designed for synthetic use. The reactor is equipped with a magnetic stirrer as well as a plethora of pressure, temperature, and power controls. The maximum operating pressure of the reactor is 2 × 10^6^ Pa. The power and temperature ranges are 15 W to 300 W and 60°C to 250 °C, respectively.

### 4.2. Synthesis

#### 4.2.1. General Procedures for the Synthesis of Imidazolium Halides **1**–**14** Using Conventional Preparation (CP)

For a solution of 1-hexylimidazole (1 eq) in toluene, the appropriate alkyl halide (1.1 eq) was added at room temperature, which was followed by stirring at 80 °C for 18 h. The completion of the reaction was marked by the separation of viscous liquid from the initially obtained clear and homogenous mixture of 1-hexylimidazole and alkyl halide in toluene. The product was isolated by extraction to remove the unreacted starting materials and solvent. Subsequently, the ionic liquid was washed with ethyl acetate. In each case, a small amount of ethanol was added to the IL, which was dried at a reduced pressure to get rid of all the volatile organic compounds and water traces.

#### 4.2.2. General Procedure for the Synthesis of Imidazoluim Halides **1**–**14** under Microwave Irradiation (MW)

1-Hexylimidazole (1 eq) and the appropriate alkyl halide (1 eq) were placed in a closed vessel and exposed to irradiation for 20 min at 80 °C using a microwave irradiation. The product was then collected as described in the conventional procedure outlined earlier.

### 4.3. Characterization of Imidazolium Based Ionic Liquids Derivatives ***1***–***14***

*1-Hexyl-3-phenethyl-1H-imidazol-3-ium bromide* (**1**). It was obtained using a yellow viscous liquid. FT-IR (NaCl), cm^−1^: υ = 1560 (C=N), 2910 and 2980 (Ar-H). H-NMR (400 MHz, DMSO-*d*_6_): δH = 0.83 (t, 3H, CH_3_), 1.21 (m, 6H, CH_2_), 1.70 (quin, 2H, CH_2_), 3.18 (t, 2H, CH_2_), 4,15 (t, 2H, CH_2_), 4.51 (t, 2H, CH_2_), 7–15 (d, 2H, Ar-H), 7.10–7.24 (m, 5H, Ar-H), 7.88 (d, 1H, Ar-H), 7.93 (d, 1H, Ar-H), 9.44 (s, 1H, Ar-H), C-NMR (100 MHz, DMSO-*d*_6_): δC = 14.3 (CH_3_), 22.3 (CH_2_), 25.5 (CH_2_), 29.8 (CH_2_), 31.0 (CH_2_), 35.8 (CH_2_), 49.2 (CH_2_), 50.2 (CH_2_), 122.8 (CH), 122.9 (CH), 127.2 (CH), 128.9 (CH), 129.2 (CH), 136.5 (CH), 137.3 (C), MS [M − Br]^+^ 257.26 found for C_17_H_25_N_2_^+^, (Found: C, 60.48, H, 7.49, N, 8.27%. Calcd. for C_17_H_25_BrN_2_, C, 60.53, H, 7.47, N, 8.31%).

*1-Hexyl-3-(3-phenylpropyl)-1H-imidazol-3-ium bromide* (**2**). It was obtained using a yellow viscous liquid. FT-IR (NaCl), cm^−1^: υ = 1560 (C=N), 2910 and 2980 (Ar-H). H-NMR (400 MHz, DMSO-*d*_6_): δH = 0.83 (t, 3H, CH_3_), 1.24 (m, 6H, CH_2_), 1.79 (quin, 2H, CH_2_), 2.15 (quin, 2H, CH_2_), 2.61 (t, 2H, CH_2_), 4.20 (t, 2H, CH_2_), 4.26 (t, 2H, CH_2_), 7.20–727 (m, 5H, Ar-H), 7.91–7.93 (m, 2H, Ar-H), 9.51 (s, 1H, Ar-H) C-NMR (100 MHz, DMSO-*d*_6_): δC = 14.3 (CH_3_), 22.4 (CH_2_), 25.6 (CH_2_), 29.8 (CH_2_), 31.0 (CH_2_), 31.4 (CH_2_), 32.2 (CH_2_), 49.1 (CH_2_), 49.3 (CH_2_), 122.9 (CH), 123.1 (CH), 126.5 (CH), 128.7 (CH), 128.8 (CH), 136.6 (CH), 141.0 (C), MS [M − Br]^+^ 271.33 found for C_18_H_27_N_2_^+^,(Found: C, 61.47, H, 7.50, N, 7.91%. Calcd. for C_18_H_27_BrN_2_, C, 61.54, H, 7.57, N, 7.97%).

*3-(2-Ethoxy-2-oxoethyl)-1-hexyl-1H-imidazol-3-ium chloride* (**3**). It was obtained using a brown viscous liquid. FT-IR (NaCl), cm^−1^: υ = 1210 (C-O), 1550 (C=N), 1750 (C=O), 2850 and 2910 (Ar-H). H-NMR (400 MHz, DMSO-*d*_6_): δH = 0.85 (t, 3H, CH_3_), 1.22 (t, 3H, CH_3_), 1.26 (m, 6H, CH_2_), 1.79 (quin, 2H, CH_2_), 4.21 (q, 2H, CH_2_), 4.27 (t, 2H, CH_2_), 5.36 (s, 2H, CH_2_), 7.85 (d, 1H, Ar-H), 7.92 (d, 1H, Ar-H), 9.48 (s, 1H, Ar-H), C-NMR (100 MHz, DMSO-*d*_6_): δC = 14.3 (CH_3_), 14.4 (CH_3_), 22.3 (CH_2_), 25.5 (CH_2_), 29.8 (CH_2_), 31.0 (CH_2_), 49.4 (CH_2_), 50.0 (CH_2_), 62.30 (CH_2_), 122.6 (CH), 124.4 (CH), 137.9 (CH), 167.4 (C=O), MS [M − Cl]^+^ 239.33 found for C_13_H_23_N_2_O_2_^+^, (Found: C, 56.75, H, 8.42, N, 10.16%. Calcd. for C_13_H_23_ClN_2_ O_2_, C, 56.82, H, 8.44, N, 10.19%).

*3-(5-Ethoxy-5-oxopentyl)-1-hexyl-1H-imidazol-3-ium bromide* (**4**). It was obtained using a yellow viscous liquid. FT-IR (NaCl), cm^−1^: υ = 1150 (C-O), 1550 (C=N), 1735 (C=O), 2910 and 2950 (Ar-H). H-NMR (400 MHz, DMSO-*d*_6_): δH = 0.76 (t, 3H, CH_3_), 1.10 (t, 3H, CH_3_), 1.18 (m, 6H, CH_2_), 1.47 (quin, 2H, CH_2_), 1.84 (m, 4H, CH_2_), 2.29 (t, 2H, CH_2_), 3.98 (q, 2H, CH_2_), 4.25 (t, 2H, CH_2_), 4.30 (t, 2H, CH_2_), 8.03 (m, 2H, Ar-H), 9.77 (s, 1H, Ar-H), C-NMR (100 MHz, DMSO-*d*_6_): δC = 14.1 (CH_3_), 14.4 (CH_3_), 21.3 (CH_2_), 22.3 (CH_2_), 25.6 (CH_2_), 29.3 (CH_2_), 29.9 (CH_2_), 31.0 (CH_2_), 33.2 (CH_2_), 48.8 (CH_2_), 49.2 (CH_2_), 60.1 (CH_2_), 122.8 (CH), 122.9 (CH), 136.6 (CH), 172.9 (C=O), MS [M − Br]^+^ 281.23 found for C_16_H_29_N_2_O_2_^+^, (Found: C, 53.15, H, 8.13, N, 7.69%. Calcd. for C_16_H_29_BrN_2_ O_2_, C, 53.19, H, 8.09, N, 7.75%).

*1-Hexyl-3-(6-methoxy-6-oxohexyl)-1H-imidazol-3-ium bromide* (**5**). It was obtained using a yellow viscous liquid. FT-IR (NaCl), cm^−1^: υ = 1550 (C=N), 1735 (C=O), 2220 (CN), 2910 and 2950 (Ar-H). H-NMR (400 MHz, DMSO-*d*_6_): δH = 0.75 (t, 3H, CH_3_), 1.18–1.23 (m, 12H, CH_2_), 1.49 (quin, 2H, CH_2_), 1.79 (quin, 2H, CH_2_), 2.22 (t, 2H, CH_2_), 3.50 (s, 3H, CH_3_), 4.26 (t, 2H, CH_2_), 8.01 (m, 2H, Ar-H), 9.77 (d, 1H, Ar-H), C-NMR (100 MHz, DMSO-*d*_6_): δC = 14.1 (CH_3_), 22.3 (CH_2_), 24.1 (CH_2_), 25.3 (CH_2_), 25.6 (CH_2_), 29.6 (CH_2_), 29.9 (CH_2_), 31.0 (CH_2_), 33.4 (CH_2_), 49.0 (CH_2_), 49.2 (CH_3_), 51.5 (CH_3_), 122.8 (CH), 136.5 (CH), 173.4 (C=O), MS [M − Br]^+^ 281.50 found for C_16_H_29_N_2_O_2_^+^, (Found: C, 53.14, H, 8.11, N, 7.70%. Calcd. for C_16_H_29_BrN_2_ O_2_, C, 53.19, H, 8.09, N, 7.75%).

*3-(4-Acetoxybutyl)-1-hexyl-1H-imidazol-3-ium chloride* (**6**). It was obtained using a dark brown viscous liquid. FT-IR (NaCl), cm^−1^: υ = 1210 (C-O), 1550 (C=N), 1735 (C=O), 2910 and 2950 (Ar-H). H-NMR (400 MHz, DMSO-*d*_6_): δH = 0.60 (t, 3H, CH_3_), 1.05 (m, 6H, CH_2_), 1.45 (quin, 2H, CH_2_), 1.66 (quin, 2H, CH_2_), 1.78 (s, 3H, CH_3_), 3.25 (quin, 2H, CH_2_), 3.84 (t, 2H, CH_2_), 4.08 (t, 2H, CH_2_), 4.20 (t, 2H, CH_2_), 7.35 (d, 1H, Ar-H), 7.51 (d, 1H, Ar-H), 10.05 (s, 1H, Ar-H); C-NMR (100 MHz, DMSO-*d*_6_): δC = 13.7 (CH_3_), 20.7 (CH_3_), 22.1 (CH_2_), 25.1 (CH_2_), 25.6 (CH_2_), 26.8 (CH_2_), 29.9 (CH_2_), 30.8 (CH_2_), 49.1 (CH_2_), 49.8 (CH_2_), 63.1 (CH_2_), 122.1 (CH), 122.4 (CH), 136.7 (CH), 170.9 (C=O), MS [M − Cl]^+^ 267.18 found for C_15_H_27_N_2_O_2_^+^, (Found: C, 59.45, H, 9.03, N, 9.20%. Calcd. for C_15_H_27_ClN_2_ O_2_, C, 59.49, H, 8.99, N, 9.25%).

*1-Hexyl-3-(2-hydroxythyl)-1H-imidazol-3-ium bromide* (**7**). It was obtained through a dark brown viscous liquid. FT-IR (NaCl), cm^−1^: υ = 1580 (C=N), 2910 and 2950 (Ar-H), 3310 (O-H). H-NMR (400 MHz, CDCl_3_): δ_H_ = 0.77 (t, 3H, CH_3_), 1.21 (m, 6H, CH_2_), 1.75–1.77 (m, 2H, CH_2_), 3.81 (t, 2H, CH_2_), 4.09 (t, 2H, CH_2_), 4.28 (t, 2H, CH_2_), 4.84 (s, 1H,OH), 7.29 (s,1H,Ar-H), 7.53 (s,1H,Ar-H), 9.30 (s, 1H, Ar-H). C-NMR (100 MHz, CDCl_3_): δ_C_ = 13.8 (CH_3_), 22.2 (CH_2_), 25.7 (CH_2_), 29.9 (CH_2_), 31 (CH_2_), 49.8 (CH_2_), 52.1 (CH_2_), 59.9 (CH_2_), 122.6 (CH), 123.2 (CH), 136.4 (CH), MS [M − Br]^+^ 197.06 found for C_11_H_21_N_2_O^+^, (Found: C, 47.60, H, 7.61, N, 10.05%. Calcd. for C_11_H_21_BrN_2_ O, C, 47.66, H, 7.64, N, 10.11%).

*1-Hexyl-3-(3-hydroxypropyl)-1H-imidazol-3-ium bromide* (**8**). It was obtained using a brown viscous liquid. FT-IR (NaCl), cm^−1^: υ = 1560 (C=N) 2910 and 2950 (Ar-H), 3390 (O-H). H-NMR (400 MHz, CDCl_3_): δH = 0.68 (t, 3H, CH_3_), 1.12 (m, 6H, CH_2_), 1.73 (quin, 2H, CH_2_), 1.97 (quin, 2H, CH_2_), 3.42 (t, 2H, CH_2_), 4.11 (t, 2H, CH_2_), 4.34 (t, 2H, CH_2_), 7.37 (d, 1H, Ar-H), 7.66 (d, 1H, Ar-H), 9.72 (s, 1H, Ar-H); C-NMR (100 MHz, CDCl_3_): δC = 13.8 (CH_3_), 22.2 (CH_2_), 25.7 (CH_2_), 30.0 (CH_2_), 30.9 (CH_2_), 32.6 (CH_2_), 46.8 (CH_2_), 49.9 (CH_2_), 57.1 (CH_2_), 122.0 (CH), 123.0 (CH), 136.3 (CH), MS [M − Br]^+^ 211.32 found for C_12_H_23_N_2_O^+^, (Found: C, 49.42, H, 8.01, N, 9.55%. Calcd. for C_12_H_23_BrN_2_ O, C, 49.49, H, 7.96, N, 9.62%).

*1-Hexyl-3-(2-(2-hydroxyethoxy)ethyl)-1H-imidazol-3-ium chloride* (**9**). It was obtained through a brown viscous liquid. FT-IR (NaCl), cm^−1^: υ = 1150 (C-O), 1550 (C=N), 3400 (O-H), 2910 and 2950 (Ar-H). H-NMR (400 MHz, DMSO-*d*_6_): δH = 0.83 (t, 3H, CH_3_), 1.24 (t, 6H, CH_2_), 1.78 (quin, 2H, CH_2_), 3.45 (m, 4H, CH_2_), 3.77 (t, 2H, CH_2_), 4.22 (t, 2H, CH_2_), 4.39 (t, 2H, CH_2_), 5.77(s, 1H, OH), 7.66 (s,1H, Ar-H), 7.88 (s,1H, Ar-H), 9.28 (s, 1H, CH), C-NMR (100 MHz, DMSO-*d*_6_): δ C = 14.3 (CH_3_), 22.3 (CH_2_), 25.5 (CH_2_), 25.6 (CH_2_), 29.8 (CH_2_), 29.9 (CH_2_), 31,0 (CH_2_), 48.8 (CH_2_), 49.2 (CH_2_), 60.4 (CH_2_), 122.3 (CH), 123.3 (CH), 135.5 (CH), MS [M − Cl]^+^ 241.04 found for C_13_H_25_N_2_O_2_^+^, (Found: C, 56.38, H, 9.08, N, %. 10.10. Calcd. for C_13_H_25_ClN_2_ O_2_, C, 56.41, H, 9.10, N, 10.12%).

*1-Hexyl-3-(2-methoxyethyl)-1H-imidazol-3-ium bromide* (**10**). It was obtained using dark brown viscous liquid. FT-IR (NaCl), cm^−1^: υ = 1150 (C-O), 1550 (C=N), 2910 and 2950 (Ar-H). H-NMR (400 MHz, CDCl_3_): δH = 0.64 (t, 3H, CH_3_), 1.09 (m, 6H, CH_2_), 1.70 (quin, 2H, CH_2_), 3.15 (s, 3H, CH_3_), 3.58 (t, 2H, CH_2_), 4.13 (t, 2H, CH_2_), 4.40 (t, 2H, CH_2_), 7.41(d, 1H, Ar-H), 7.51 (d, 1H, Ar-H), 9.73 (s, 1H, Ar-H), C-NMR (100 MHz, CDCl_3_): δC = 13.7 (CH_3_), 22.2 (CH_2_), 25.6 (CH_2_), 30.0 (CH_2_), 30.9 (CH_2_), 49.6 (CH_2_), 49.9 (CH_2_), 58.8 (CH_3_), 70.1 (CH_2_), 121.8 (CH), 123.2 (CH), 136.4 (CH), MS [M − Br]^+^ 211.07 found for C_12_H_23_N_2_O^+^_,_ (Found: C, 49.45, H, 8.03, N, 9.58%. Calcd. for C_12_H_23_BrN_2_ O, C, 49.49, H, 7.96, N, 9.62%).

*3-(2-Ethoxyethyl)-1-hexyl-1H-imidazol-3-ium chloride* (**11**). It was obtained using a dark brown viscous liquid. FT-IR (NaCl), cm^−1^: υ = 1110 (C-O), 1560 (C=N), 2910 and 2950 (Ar-H), 3078 (Ar-H). H-NMR (400 MHz, CDCl_3_): δH = 0.67 (t, 3H, CH_3_), 0.96 (t, 3H, CH_3_), 1.11 (m, 6H, CH_2_), 1.71 (quin, 2H, CH_2_), 3.32 (t, 2H, CH_2_), 3.62 (t, 2H, CH_2_), 4.14 (t, 2H, CH_2_), 4.40 (q, 2H, CH_2_), 7.38 (s, 1H, Ar-H), 7.49 (s, 1H, Ar-H), 9.88 (s, 1H, Ar-H), C-NMR (100 MHz, CDCl_3_): δC = 13.7 (CH_3_), 14.8 (CH_3_), 22.2 (CH_2_), 25.7 (CH_2_), 30.0 (CH_2_), 30.9 (CH_2_), 49.7 (CH_2_), 49.8 (CH_2_), 66.5 (CH_2_), 68.2 (CH_2_), 121.6 (CH), 123.1 (CH), 136.8 (CH), MS [M − Cl]^+^ 224.47 found for C_13_H_25_N_2_O^+^, (Found: C, 59.85, H, 9.69, N, 10.68%. Calcd. for C_13_H_25_ClN_2_ O, C, 59.87, H, 9.66, N, 10.74%).

*3-(5-Ethoxy-5-oxopentyl)-1-hexyl-1H-imidazol-3-ium bromide* (**12**). It was obtained using a dark brown viscous liquid. FT-IR (NaCl), cm^−1^: υ = 1250 (C-O), 1600 (C=N), 2910 and 2980 (Ar-H). H-NMR (400 MHz, DMSO-*d*_6_): δH = 0.80 (t, 3H, CH_3_), 1.14–1.20 (m, 6H, CH_2_), 1.76–1.79 (quin, 2H, CH_2_), 4.25 (t, 2H, CH_2_), 4.38 (t, 2H, CH_2_), 4.68 (t, 2H, CH_2_), 6.94–6.96 (m, 2H, Ar-H), 7.12–7.28 (m, 3H, Ar-H), 7.95 (d, 1H, Ar-H), 7.99 (d, 1H, Ar-H) 9.60 (s, 1H, Ar-H). C-NMR (100 MHz, DMSO-*d*_6_): δC = 14.2 (CH_3_), 21.5 (CH_2_), 22.3 (CH_2_), 25.5 (CH_2_), 29.9 (CH_2_), 31 (CH_2_), 49.3 (CH_2_), 66.2 (CH_2_),115.0 (CH), 121.6 (CH), 122.8 (CH), 128.6 (CH), 129.3 (CH), 129.9 (CH), 137.2 (CH),158.2 (C), MS [M − Br]^+^ 272.64 found for C_17_H_25_N_2_O^+^, (Found: C, 57.71, H, 7.10, N, 7.86%. Calcd. for C_17_H_25_BrN_2_ O, C, 57.79, H, 7.13, N, 7.93%).

*1-Hexyl-3-(3-phenoxypropyl)-1H-imidazol-3-ium bromide* (**13**). It was obtained through a yellow viscous liquid. FT-IR (NaCl), cm^−1^: υ = 1250 (C-O), 1600 (C=N), 2910 and 2980 (Ar-H). H-NMR (400 MHz, DMSO-*d*_6_): δH = 0.76 (t, 3H, CH_3_), 1.14 (m, 6H, CH_2_), 1.71 (quin, 2H, CH_2_), 2.29 (quin, 2H, CH_2_), 3.98 (t, 2H, CH_2_), 4.20 (t, 2H, CH_2_), 4.45 (t, 2H, CH_2_), 6.84–6.86 (m, 3H, Ar-H), 7.20 (t, 2H, Ar-H), 7.95 (d, 1H, Ar-H), 7.99 (d, 1H, Ar-H), 9.63 (s, 1H, Ar-H). C-NMR (100 MHz, DMSO-*d*_6_): δ C = 14.2 (CH_3_), 22.3 (CH_2_), 25.6 (CH_2_), 29.6 (CH_2_), 29.9 (CH_2_), 31.0 (CH_2_), 46.9 (CH_2_), 49.3 (CH_2_), 65.0 (CH_2_), 114.8 (CH), 121.1 (CH), 122.9 (CH), 123 (CH),129.8 (CH), 136.6 (CH), 158.5 (C), MS [M − Br]^+^ 286.80 found for C_18_H_27_N_2_O^+^, (Found: C, 58.79, H, 7.39, N, 7.59%. Calcd. for C_18_H_27_BrN_2_ O, C, 58.86, H, 7.41, N, 7.63%).

*3-(3-Cyanopropyl)-1-hexyl-1H-imidazol-3-ium chloride* (**14**). It was obtained through dark brown viscous liquid. FT-IR (NaCl), cm^−1^: υ = 1550 (C=N), 2220 (CN), 2910 and 2950 (Ar-H). H-NMR (400 MHz, DMSO-*d*_6_): δH = 0.84 (t, 3H, CH_3_), 1.25 (m, 6H, CH_2_), 1.79 (quin, 2H, CH_2_), 2.19 (quin, 2H, CH_2_), 2.65 (t, 2H, CH_2_), 4.19 (t, 2H, CH_2_), 4.33 (t, 2H, CH_2_), 7.92(d,1H, Ar-H), 7.96 (d, 1H, Ar-H), 9.67 (s, 1H, Ar-H), C-NMR (100 MHz, DMSO-*d*_6_): δC = 14.0 (CH_2_), 14.3 (CH_3_), 22.3 (CH_2_), 25.6 (CH_2_), 25.8 (CH_2_), 29.7 (CH_2_), 31.0 (CH_2_), 48.1 (CH_2_), 49.3 (CH_2_), 120.1 (C), 122.9 (CH), 123.0 (CH), 137.0 (CH), MS [M − Cl]^+^ 220.11 found for C_13_H_22_N_3_^+^, (Found: C, 60.97, H, 8.69, N, 16.38%. Calcd. for C_13_H_22_ClN_3_, C, 61.04, H, 8.67, N, 16.43%).

### 4.4. Determination of IZ, MIC, and MBC

The antimicrobial activity was initially estimated in terms of inhibition zone (IZ) measurements by using the agar disc diffusion method. The test was performed by sub-culturing 24 h fresh bacterial cultures onto the surface of Muller–Hinton agar plates. The antibacterial activity was assayed using filter paper discs (6 mm i.d.) loaded with 10 μg of a tested compound. Loaded discs were transferred onto the center of inoculated Petri-plates, which were then maintained for 2 h in a refrigerator at 4 °C to allow for the diffusion of the bioactive compound. The diameter of the inhibition zone was measured (in mm) after 24 h incubation at 37 °C. Sterile distilled water was used as a control. All compounds were tested against clinical samples of *Staphylococcus aureus*, *Bacillus cereus*, *Acinetobacter baumannii*, *Escherichia coli*, *Klebsiella pneumonia*, and *Pseudomonas aeruginosa* as test strains.

The minimum inhibitory concentration (MIC) and minimum bactericidal concentration (MBC) determined by a CLSI microdilution-based method were used to evaluate antibacterial potentials [45]. The test compound was dissolved in sterile, distilled water and diluted to a final concentration of 512 µg/mL inMueller-Hinton broth (Becton Dickinson, Franklin Lakes, NJ, USA) [46]. Two-fold serial dilutions were prepared in a 96-well micro-titer plate. Bacterial suspension containing approximately 1 × 10^8^ CFU/mL were prepared from 24 h agar plates with Mueller Hinton broth. Aliquots of 100 µL of each bacterial suspension was mixed with 100 µL serially diluted and tested compound in a microtiter plate [47]. Un-inoculated wells were prepared as control samples. Plates were incubated at 37 °C for 24 h. The MIC was defined as the lowest concentration of the test compound producing no visible growth. The MBC was determined by the transfer of aliquots from wells containing no growth on the nutrient agar plates and tested for colony formation after sub-culturing. All experiments were performed in triplicate.

## Figures and Tables

**Table 1:** Reaction conditions and yields for the synthesis of ILs **1** and **2** using conventional procedure (CP) and microwave (MW) irradiation conditions.

Compound	Alkyl Bromide RBr	Yield (%) of the Quaternization Step	
		CP	MW
**1**	Ph (CH_2_)_2_Br	71	87
**2**	Ph (CH_2_)_3_Br	72	87

**Table 2:** Reaction conditions and yields for the Synthesis of ILs **3**–**6** using conventional preparation and microwave irradiation conditions.

Compound	Alkyl Halide RX	Yield (%) of the Quaternization Step	
		CP	MW
**3**	EtO_2_CCH_2_Cl	79	87
**4**	EtO_2_C(CH_2_)_4_Br	74	88
**5**	MeO_2_C(CH_2_)_5_Br	78	89
**6**	MeCO_2_(CH_2_)_4_Cl	69	87

**Table 3:** Reaction conditions and yields for the quaternization of the 1-hexylimidazole using conventional preparation and microwave irradiation conditions.

Compound	RX	Yield (%) of the Quaternization Step	
		CP	MW
**7**	HO(CH_2_)_2_Br	78	90
**8**	HO(CH_2_)_3_Br	75	87
**9**	HO(CH_2_)_2_O(CH_2_)_2_Cl	65	81

**Table 4:** Reaction conditions and yields for the synthesis of ILs **10**–**13** using conventional preparation and microwave irradiation conditions.

Compound	RX	Yield (%) of the Quaternization Step	
		CP	MW
**10**	CH_3_O(CH_2_)_2_Br	71	86
**11**	CH_3_CH_2_O(CH_2_)_2_Cl	69	85
**12**	PhO(CH_2_)_2_Br	72	83
**13**	PhO(CH_2_)_3_Br	72	84

**Table 5:** Antibacterial activity of ILs **1**–**14** against seven bacteria expressed as MIC (mm) ^a^.

ILs	*S. aureus*	*B. cereus*	*B.* *amyloliquefaciens*	*E. coli*	*A. baumannii*	*K. pneumonia*	*P. aeruginosa*
	IZ (mm)	IZ (mm)	IZ (mm)	IZ (mm)	IZ (mm)	IZ (mm)	IZ (mm)
**1**	40	16	48	12	9	13	10
**2**	32	20	50	15	12	18	16
**3**	0	0	0	0	0	0	0
**4**	16	0	15	7	0	8	0
**5**	14	0	15	6	0	8	0
**6**	21	0	6	0	0	0	0
**7**	19	0	10	0	0	0	0
**8**	39	0	23	0	0	0	0
**9**	21	0	7	14	0	14	
**10**	20	0	11	0	0	0	6
**11**	11	0	14	0	0	8	0
**12**	35	27	51	17	16	23	20
**13**	32	21	57	14	11	17	0
**14**	0	0	0	0	0	0	0
**Amp**	29	0	30	0	0	0	0
**RD**	40	16	19	9	15	0	14
**DA**	43	22	25	0	28	0	0
**K**	26	21	32	20	0	0	23

^a^ Results are the mean of three repetitions.

**Table 6:** Antimicrobial activity of ILs **1**–**14** expressed as MIC & MBC (μg/mL) ^a^.

ILs	*S. aureus*		*B. cereus*		*B. amyloliquefaciens*		*E. coli*		*A. baumannii*		*K. pneumonia*		*P. aeruginosa*	
	MIC	MBC	MIC	MBC	MIC	MBC	MIC	MBC	MIC	MBC	MIC	MBC	MIC	MBC
**1**	4	4	64	64	4	8	64	64	128	>256	32	32	128	128
**2**	8	8	16	16	4	4	32	32	64	64	16	16	32	32
**3**	-	-	-	-	-	-	-	-	-	-	-	-	-	-
**4**	128	128	-	-	128	128	>256	>256	-	-	>256	>256	-	-
**5**	128	128	-	-	128	128	>256	>256	-	-	>256	>256	-	-
**6**	128	128	-	-	>256	>256	-	-	-	-	-	-	-	-
**7**	64	128	-	-	128	>256	-	-	-	-	-	-	-	-
**8**	4	16	-	-	32	32	-	-	-	-	-	-	-	-
**9**	64	64	-	-	>256	>256	64	64	-	-	16	32	-	-
**10**	64	64	-	-	128	128	-	-	-	-	-	-	-	-
**11**	128	>256	-	-	128	128	-	-	-	-	>256	>256	-	-
**12**	4	4	8	8	4	4	16	32	16	16	8	8	8	16
**13**	4	8	8	16	2	2	32	64	64	128	16	16	-	-
**14**	-	-	-	-	-	-	-	-	-	-	-	-	-	-
**Amp**	8	8	-	-	8	8	-	-	-		-	-	-	-
**RD**	4	4	16	16	16	16	>256	>256	32	32	-	-	32	32
**DA**	4	4	8	8	8	8	-	-	16	16	-	-	-	-
**K**	8	16	16	32	4	8	16	16	-	-	-	-	16	16

^a^ Results are the mean of three repetitions.

## Supplementary Materials

Supplementary data (^1^H-NMR, ^13^C-NMR, DEPT-^13^C-NMR, APT-^13^C-NMR, and Mass spectra of the newly synthesized ionic liquids **1**–**14**) associated with this article are available online.
