# Supplementary material for: Microwave-Assisted Synthesis of Some Potential Bioactive Imidazolium-Based Room-Temperature Ionic Liquids
Source: Molecules. 2018 Jul 15;23(7):1727. doi: 10.3390/molecules23071727 (PMC6099736; doi:10.3390/molecules23071727)
Supplement: Supplementary file 1 [file molecules-23-01727-s001.pdf]

Supporting Information

Microwave-Assisted Synthesis of Some Potential Bioactive Imidazolium-Based Room-Temperature Ionic Liquids

Ahmed H. Albalawi, Wael S. El-Sayed, Ateyatallah Aljuhani, Saud M. Almutairi, Nadjet Rezki , Mohamed R. Aouad and Mouslim Messali*

*E-mail: mouslim@mail.be

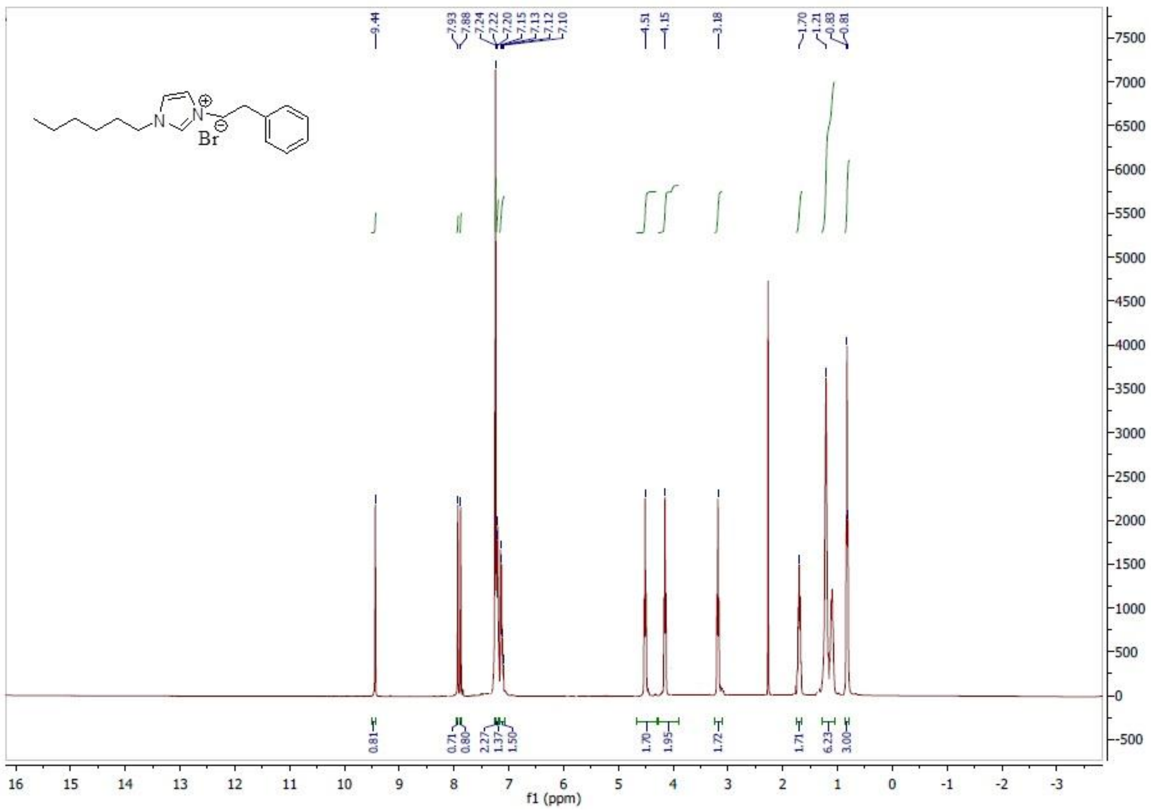

Figure S1. ^{1}H NMR spectrum of IL **1** in DMSO-d_6 (400 MHz)

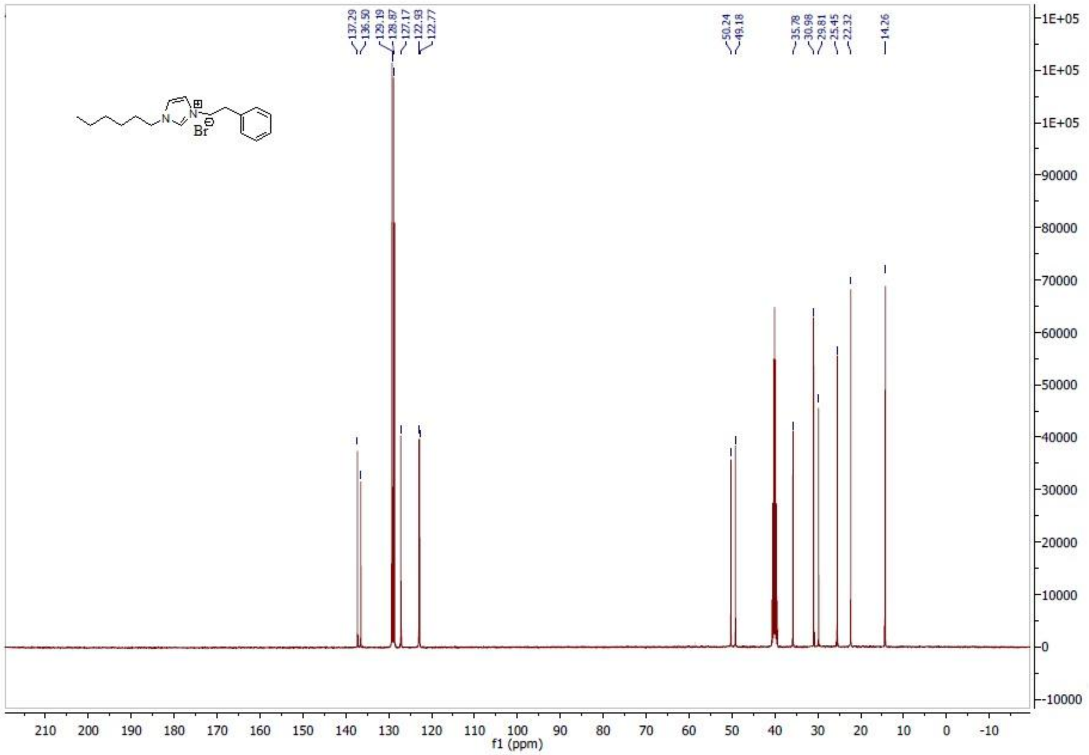

Figure S2. ^{13}C NMR spectrum of IL **1** in DMSO-d_6 (100 MHz)

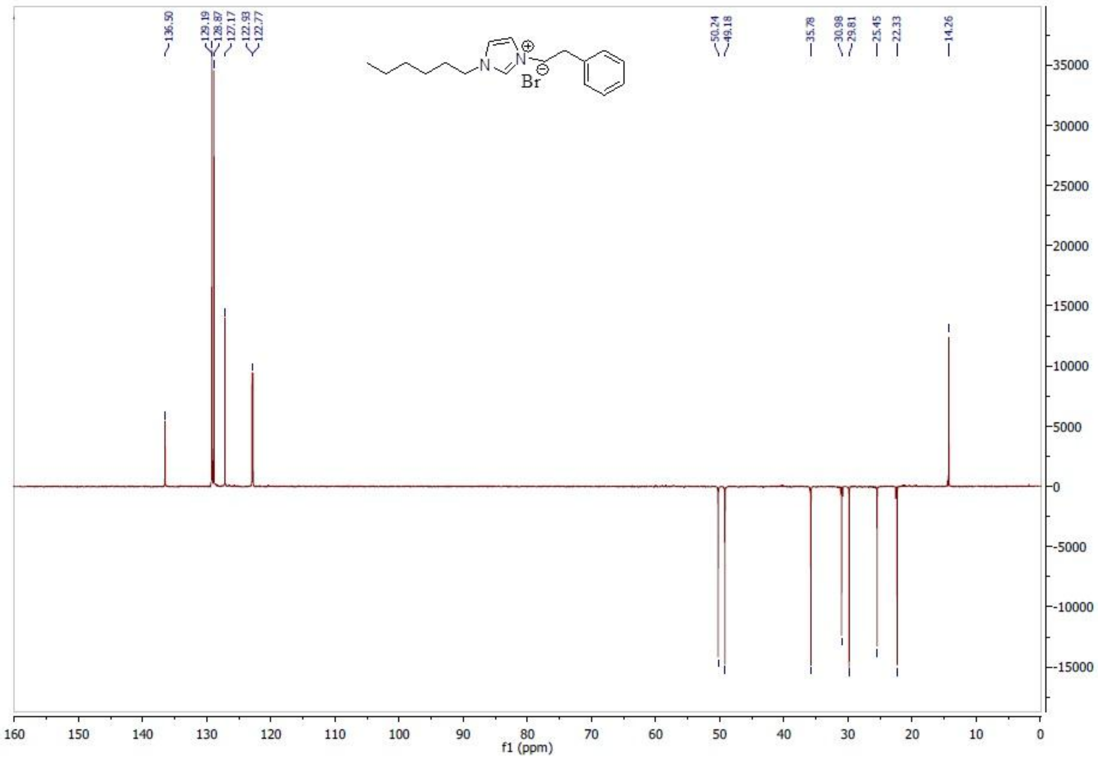

Figure S3. ^{13}C-DEPT- NMR spectrum of IL **1** in DMSO-d_6 (100 MHz)

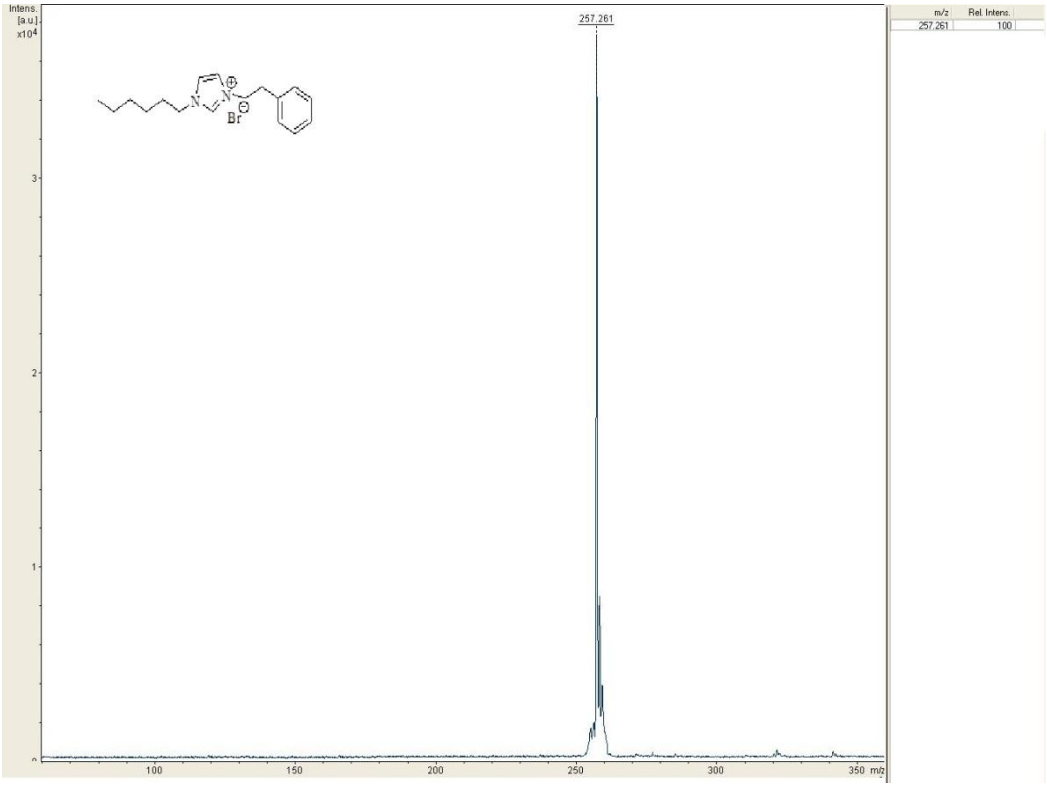

Figure S4. Mass spectrum of IL **1**

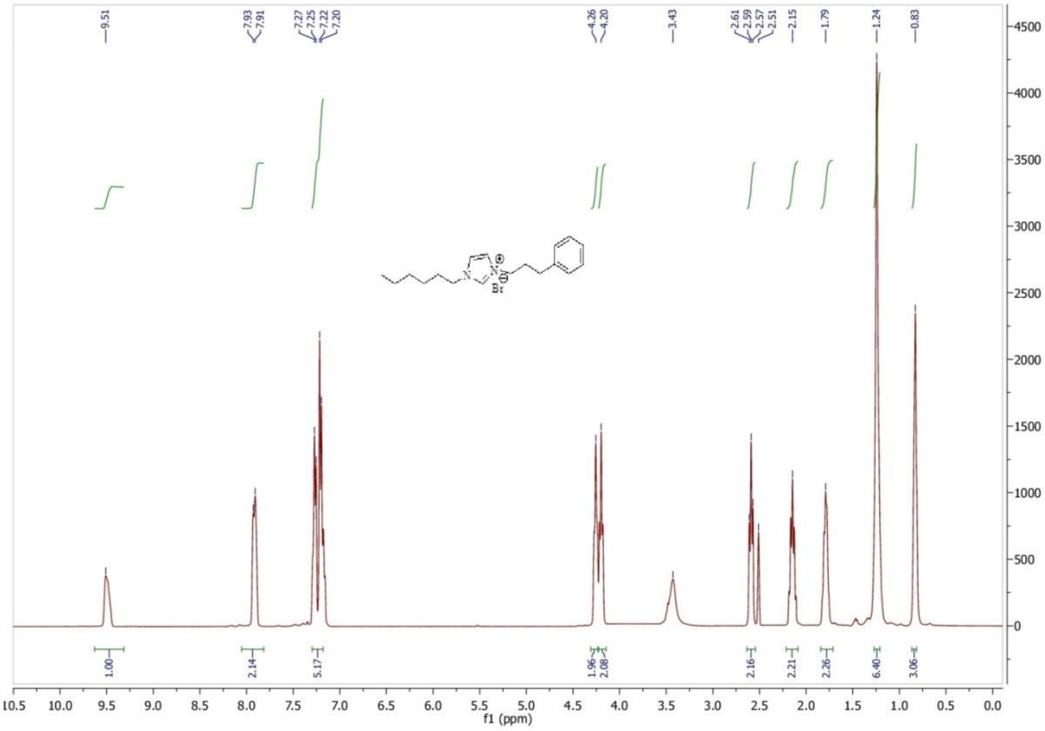

Figure S5. ^{1}H NMR spectrum of IL **2** in DMSO-d_6 (400 MHz)

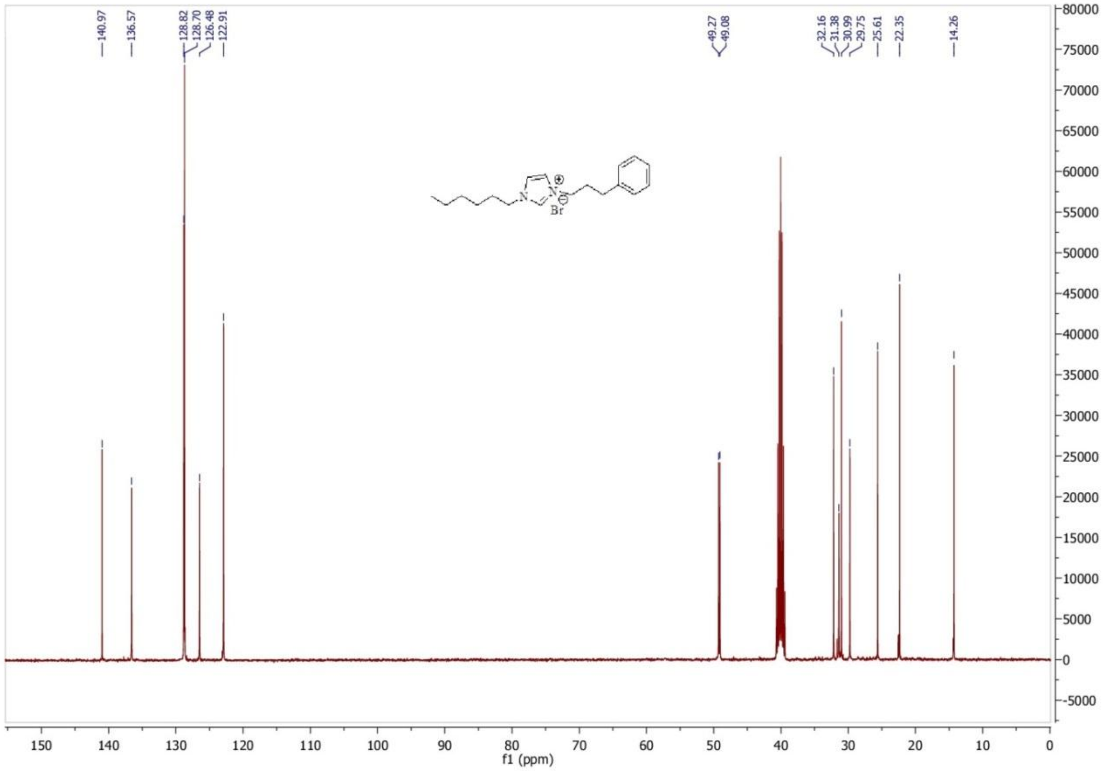

Figure S6. ^{13}C NMR spectrum of IL **2** in DMSO-d_6 (100 MHz)

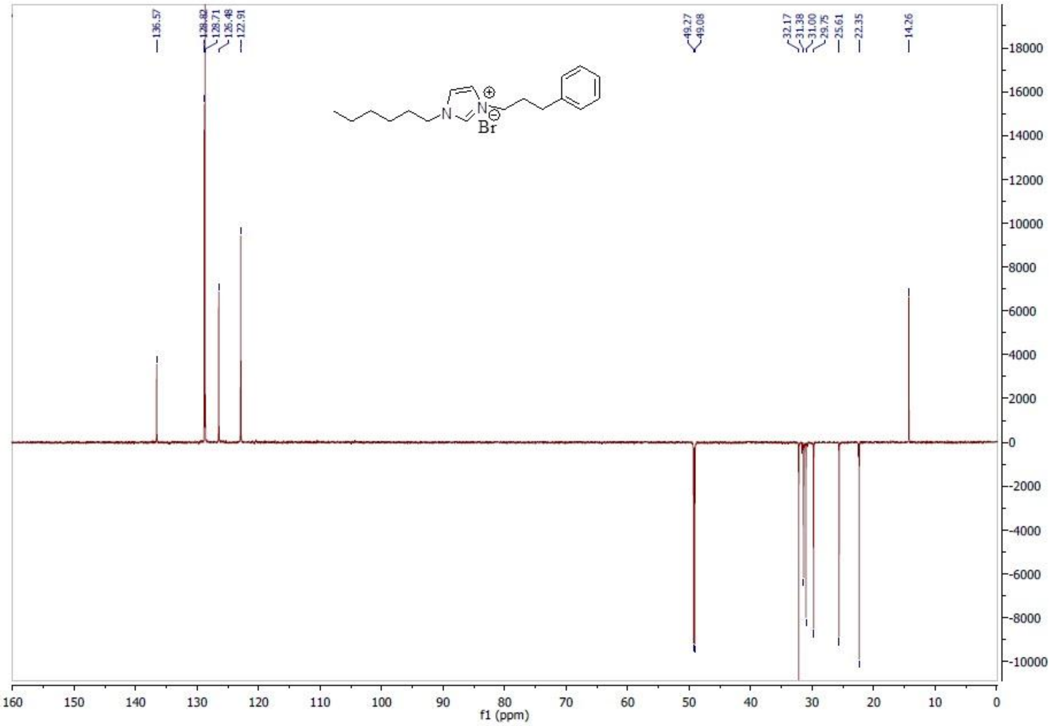

Figure S7. ^{13}C-DEPT- NMR spectrum of IL **2** in DMSO-d_6 (100 MHz)

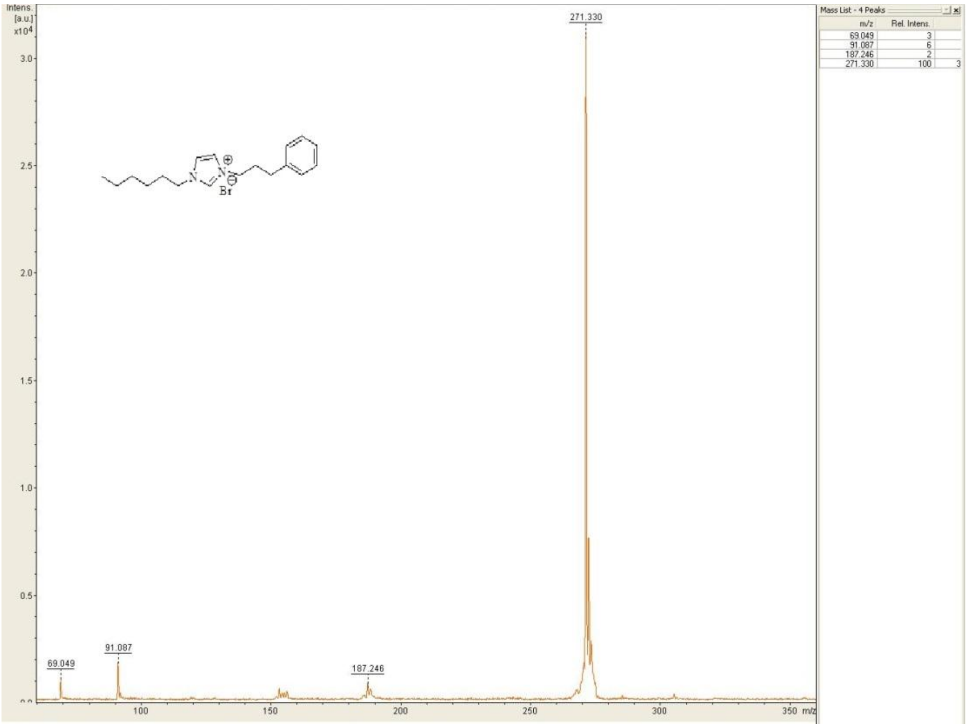

Figure S8. Mass spectrum of IL **2**

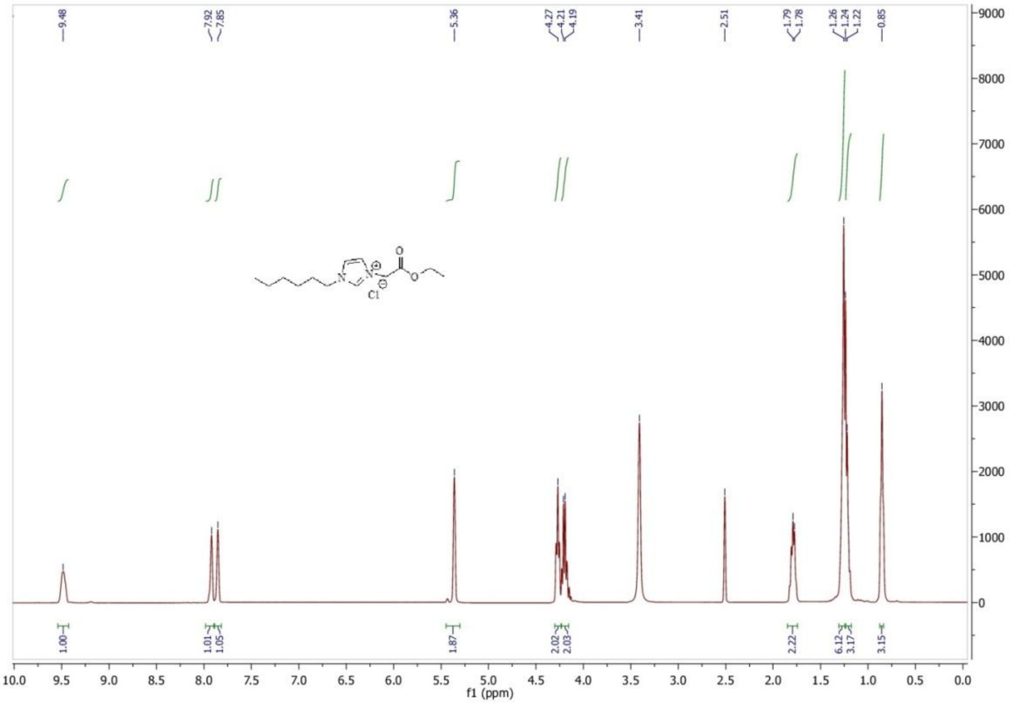

Figure S9. ^{1}H NMR spectrum of IL **3** in DMSO-d_6 (400 MHz)

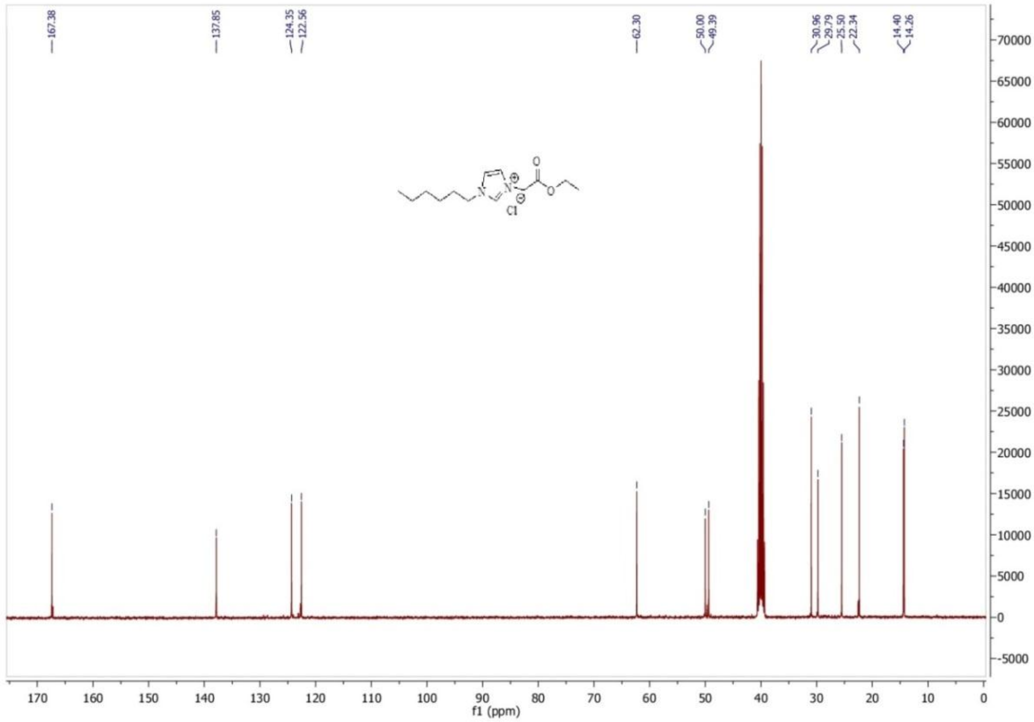

Figure S10. ^{13}C NMR spectrum of IL **3** in DMSO-d_6 (100 MHz)

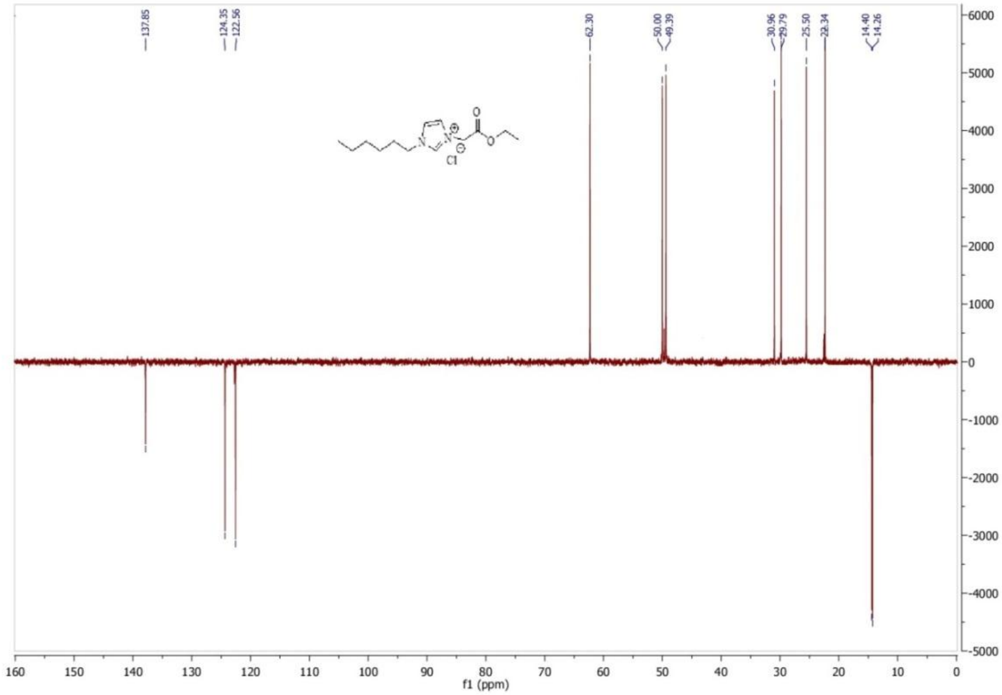

Figure S11. ^{13}C-APT- NMR spectrum of IL **3** in DMSO-d_6 (100 MHz)

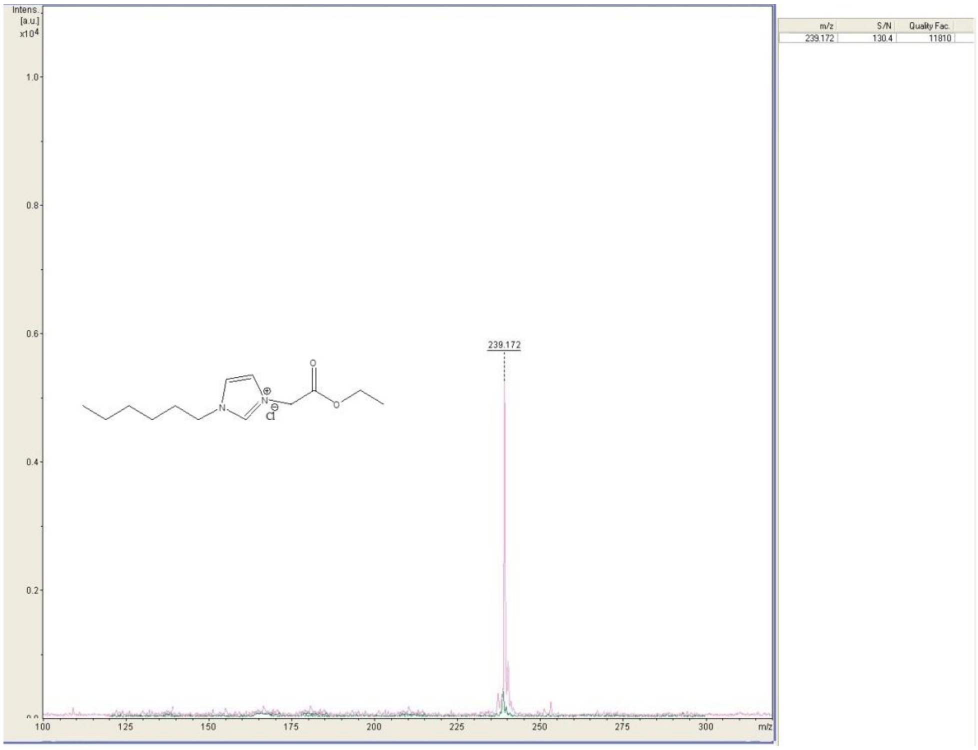

Figure S12. Mass spectrum of IL **3**

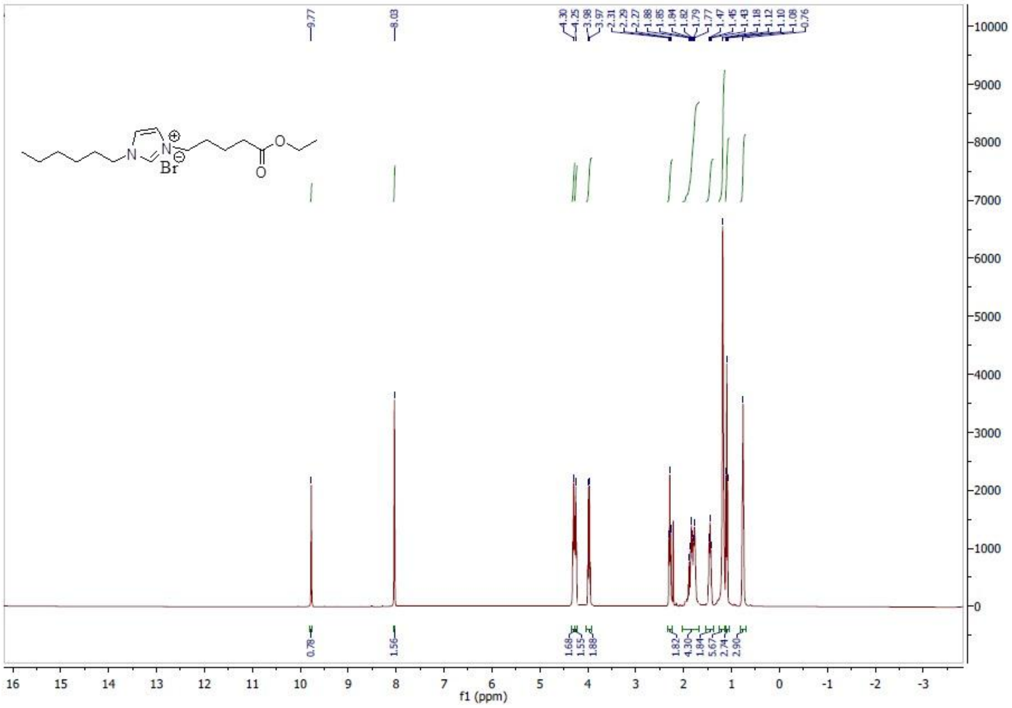

Figure S13. ^{1}H NMR spectrum of IL **4** in DMSO-d_6 (400 MHz)

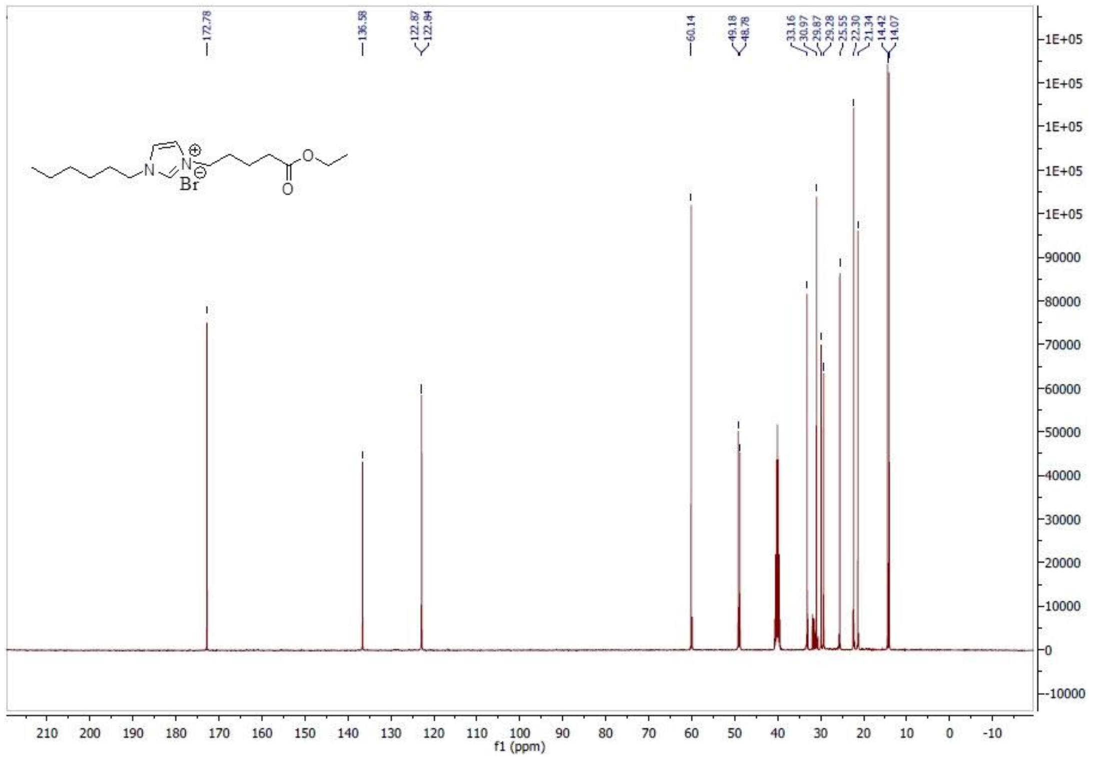

Figure S14. ^{13}C NMR spectrum of IL **4** in DMSO-d_6 (100 MHz)

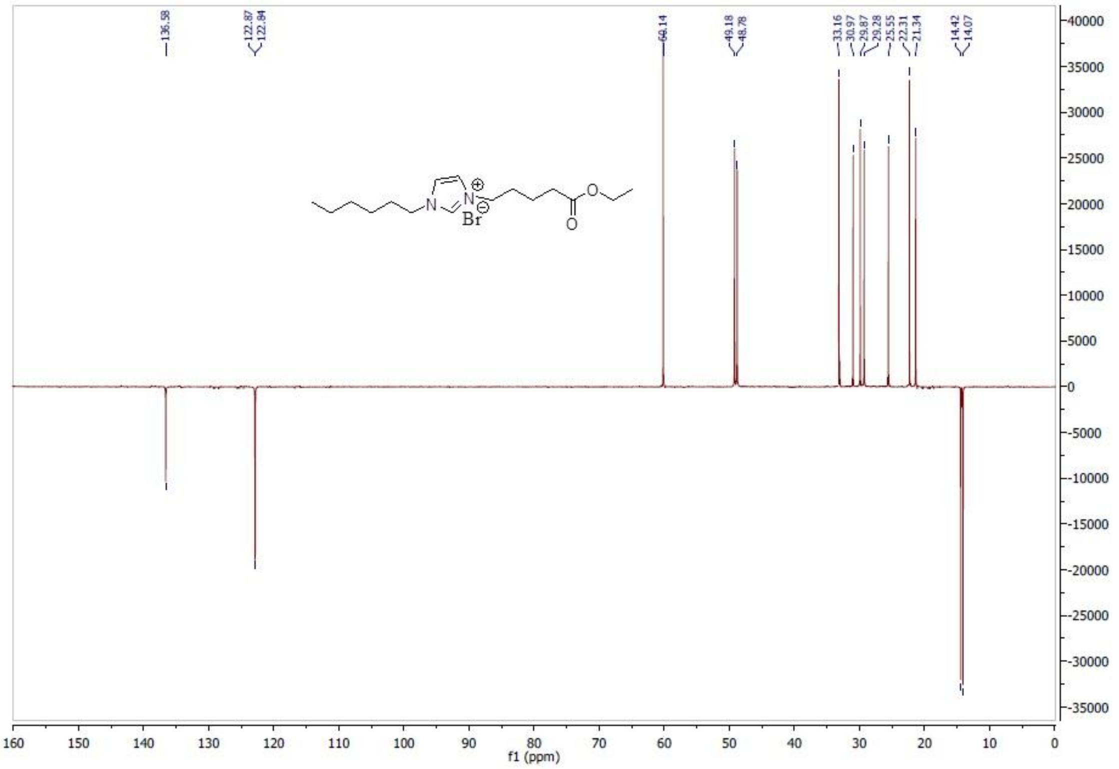

Figure S15. ^{13}C-APT- NMR spectrum of IL **4** in DMSO-d_6 (100 MHz)

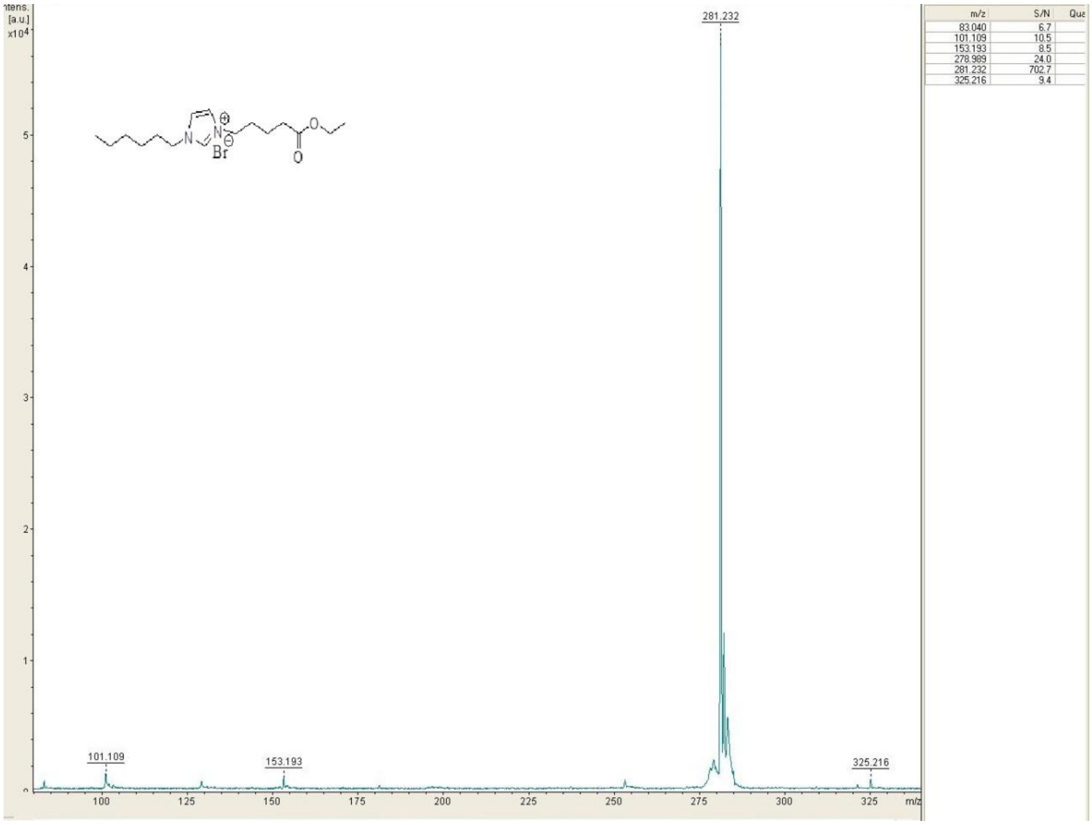

Figure S16. Mass spectrum of IL **4**

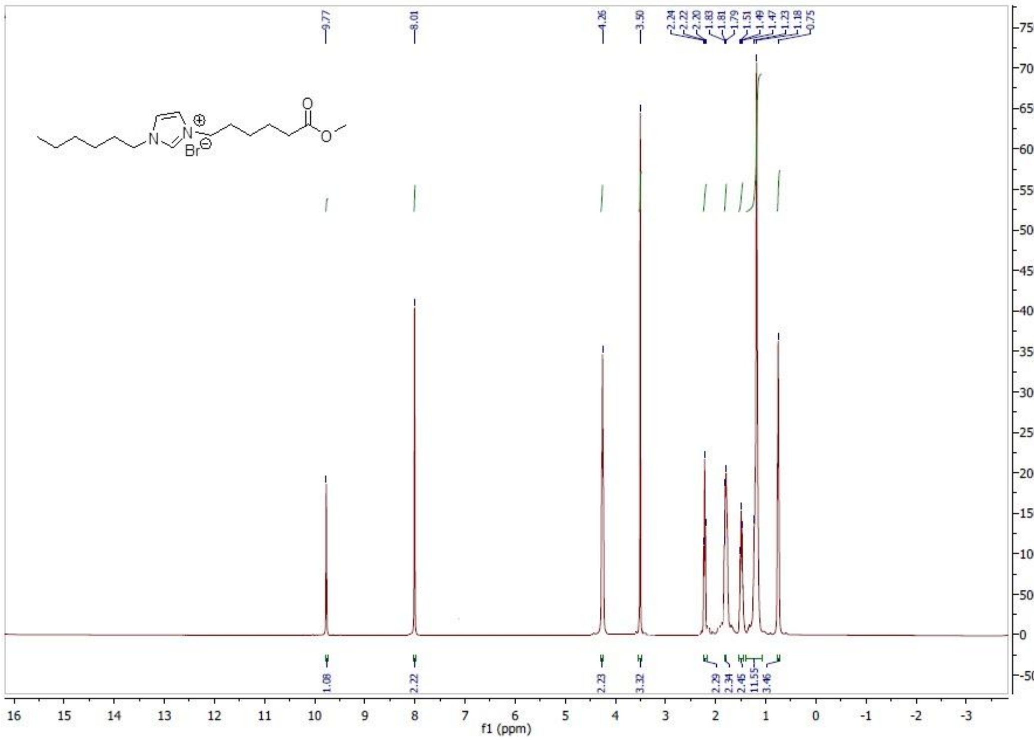

Figure S17. ^{1}H NMR spectrum of IL **5** in DMSO-d_6 (400 MHz)

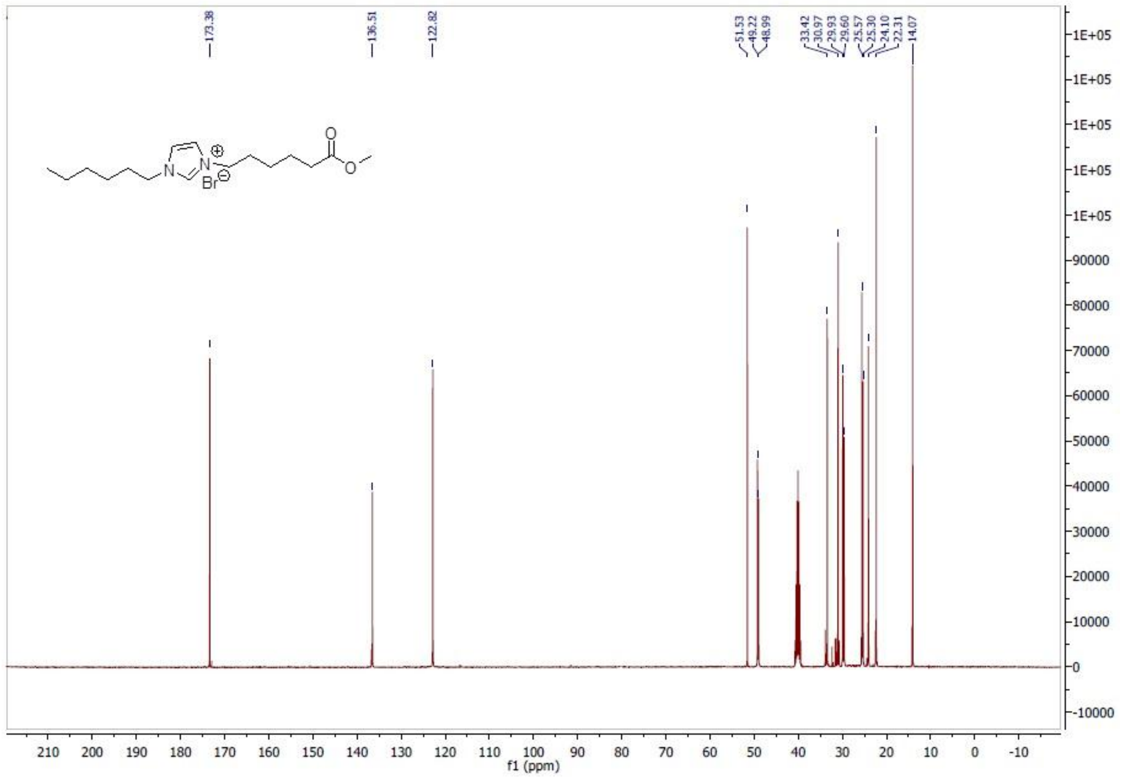

Figure S18. ^{13}C NMR spectrum of IL **5** in DMSO-d_6 (100 MHz)

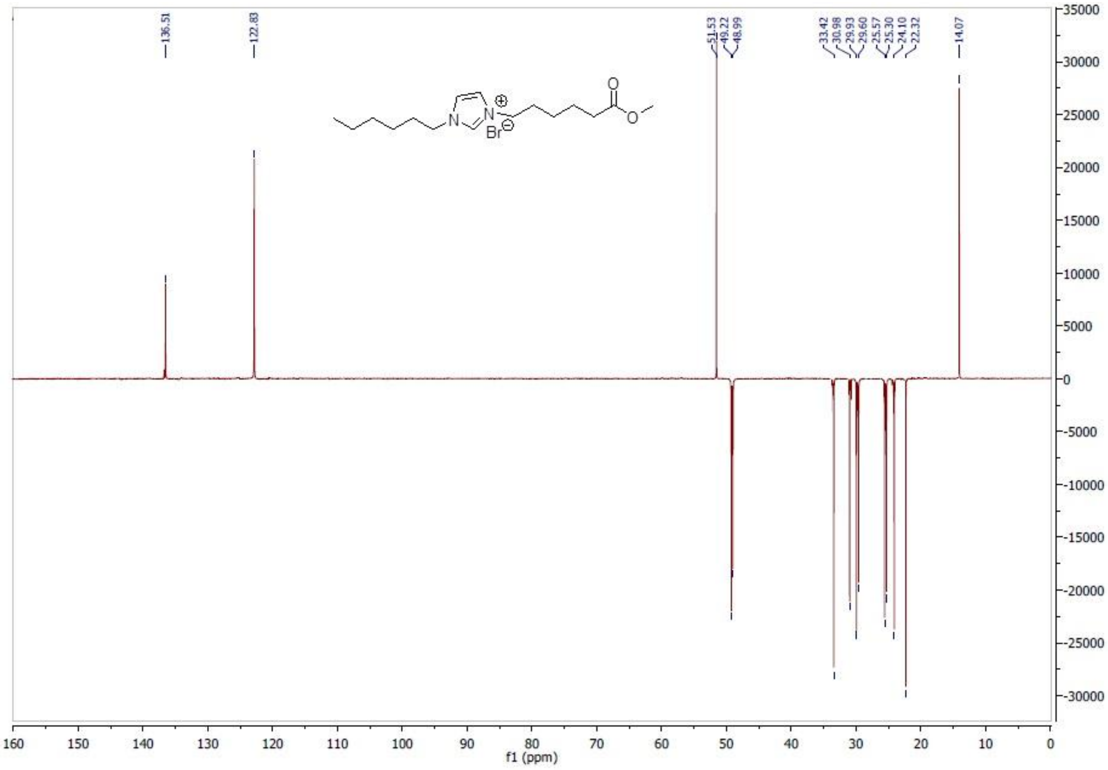

Figure S19. ^{13}C-DEPT- NMR spectrum of IL **5** in DMSO-d_6 (100 MHz)

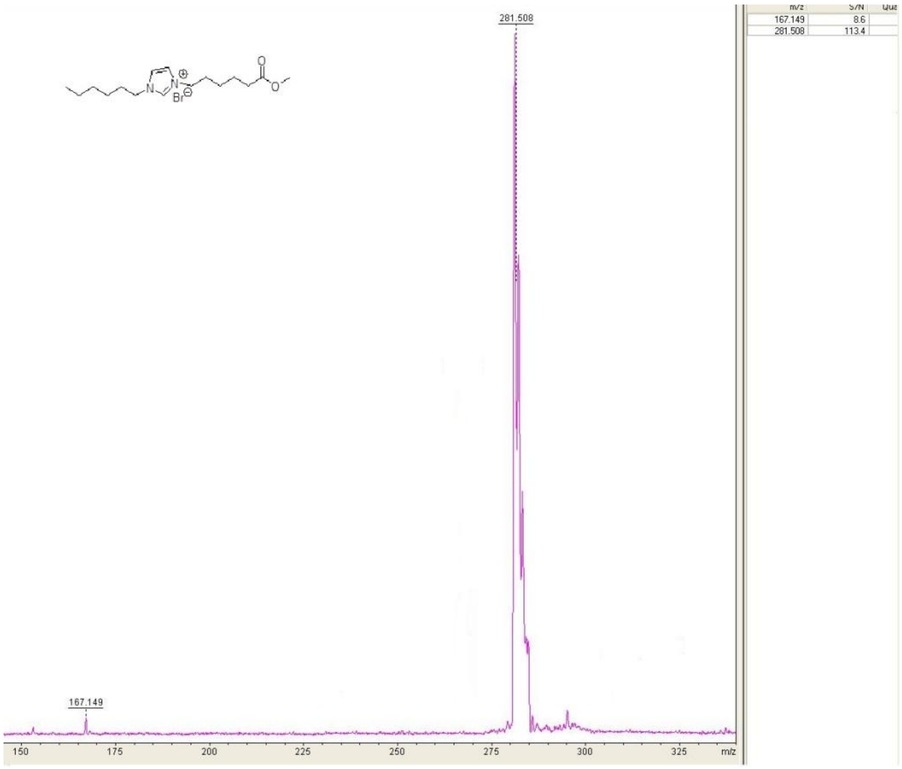

Figure S20. Mass spectrum of IL **5**

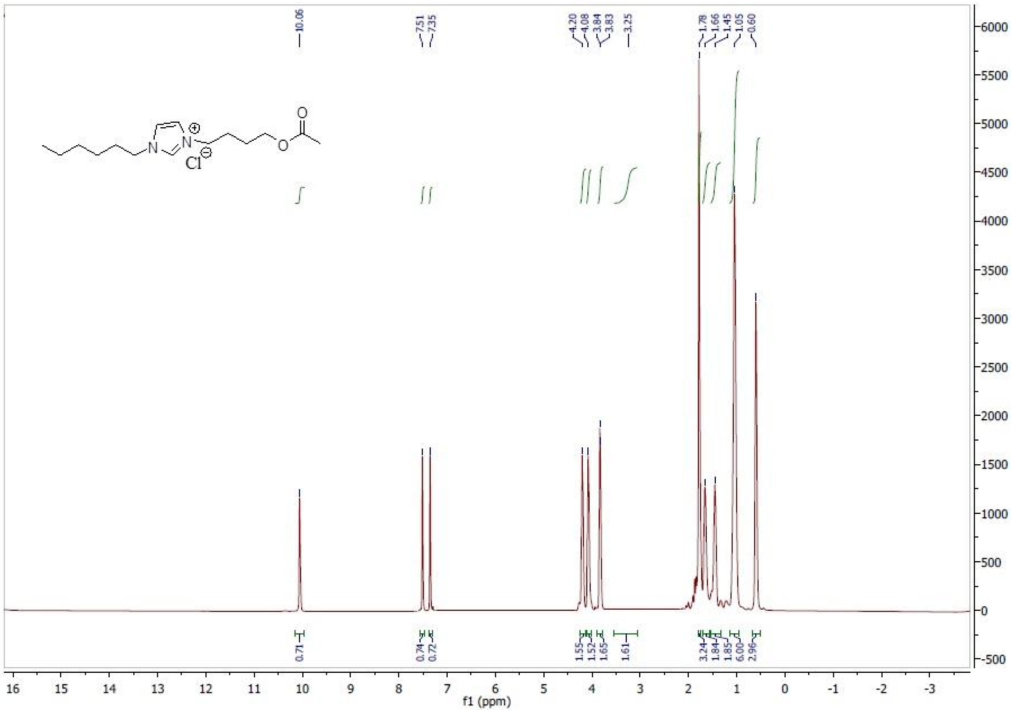

Figure S21. ^{1}H NMR spectrum of IL **6** in $CDCl_3$ (400 MHz)

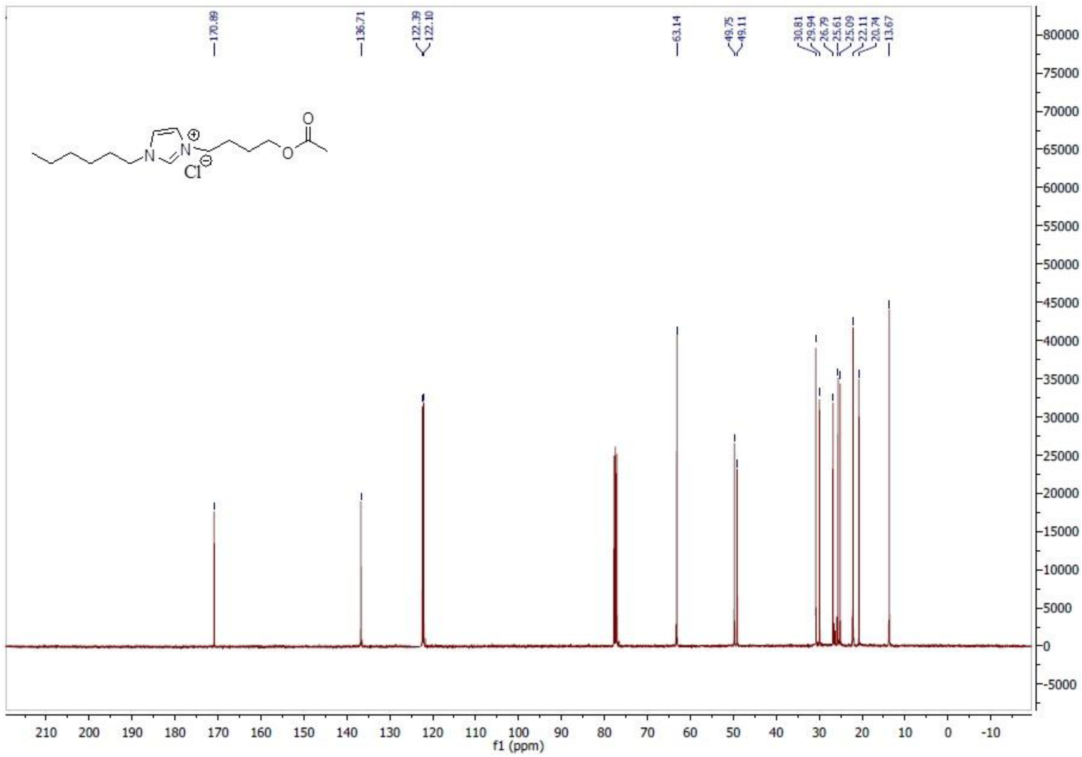

Figure S22. ^{13}C NMR spectrum of IL **6** in $CDCl_3$ (100 MHz)

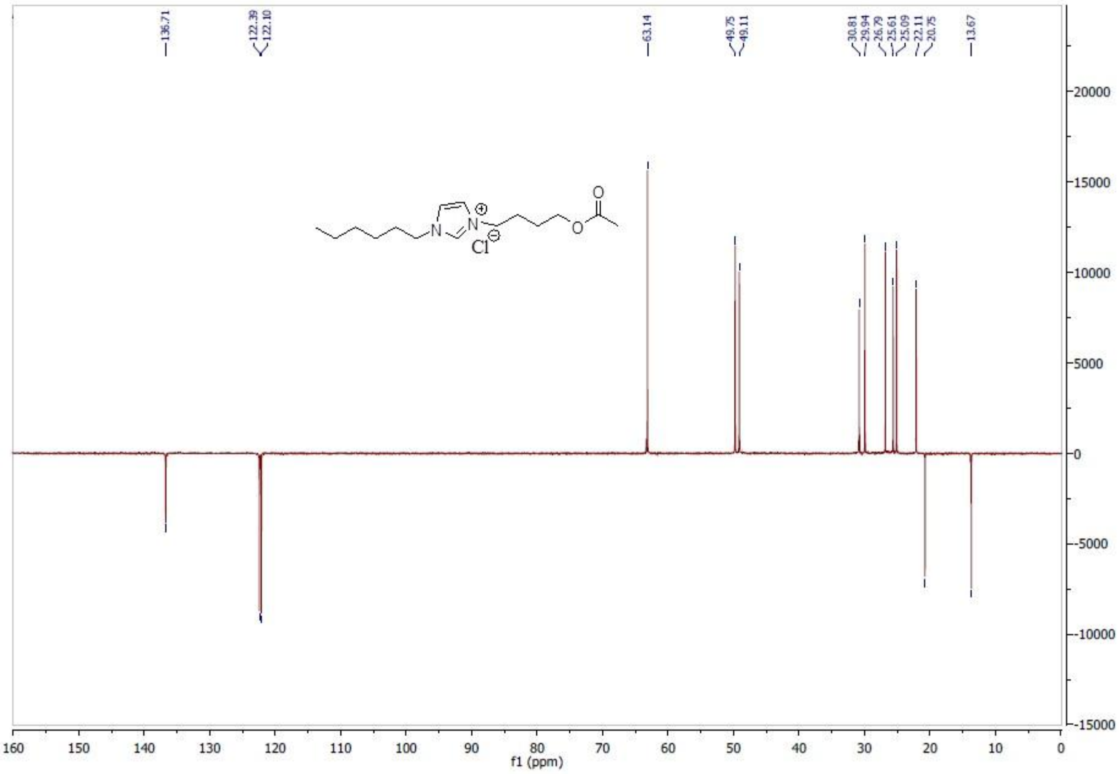

Figure S23. ^{13}C-APT- NMR spectrum of IL **6** in $CDCl_3$ (100 MHz)

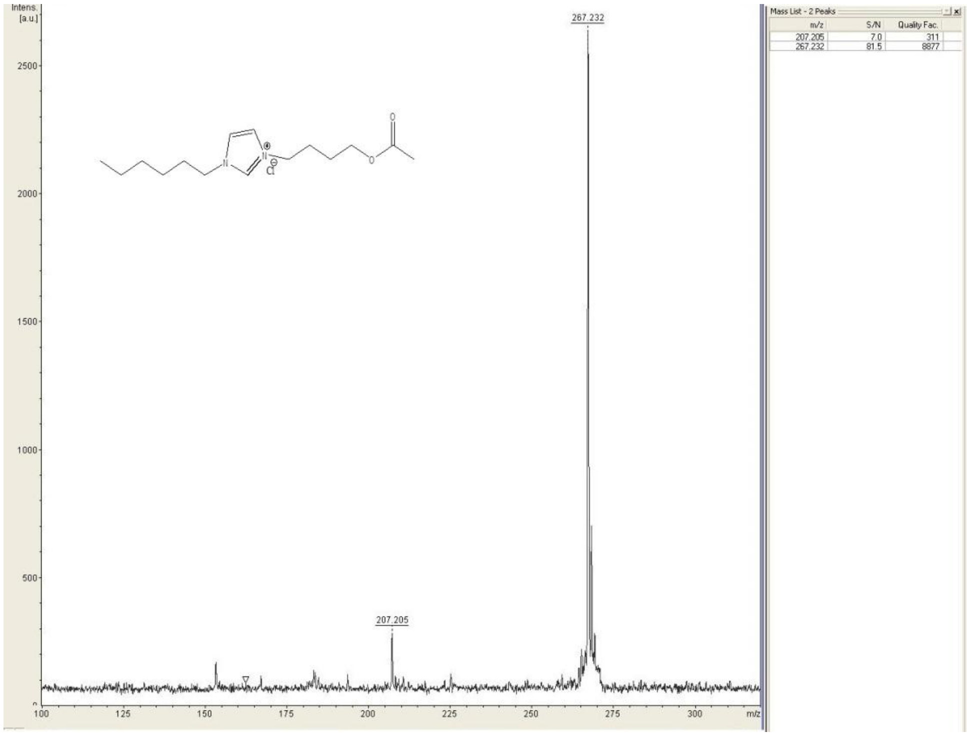

Figure S24. Mass spectrum of IL **6**

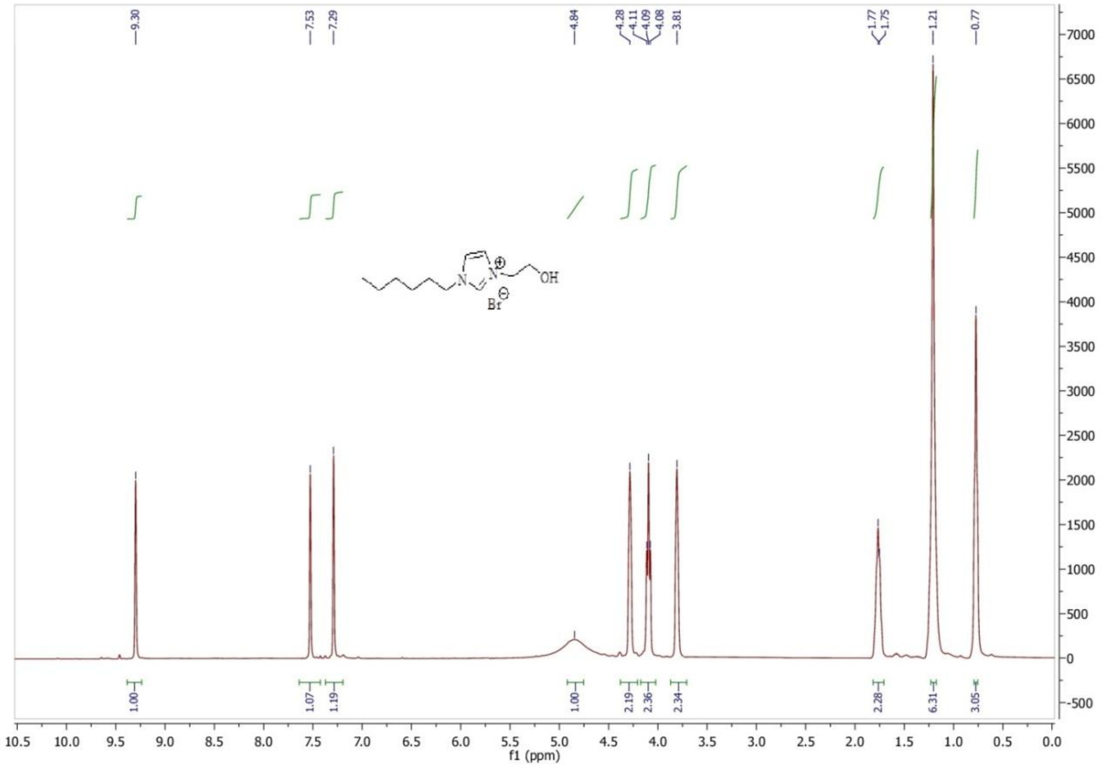

Figure S25. ^{1}H NMR spectrum of IL **7** in $CDCl_3$ (400 MHz)

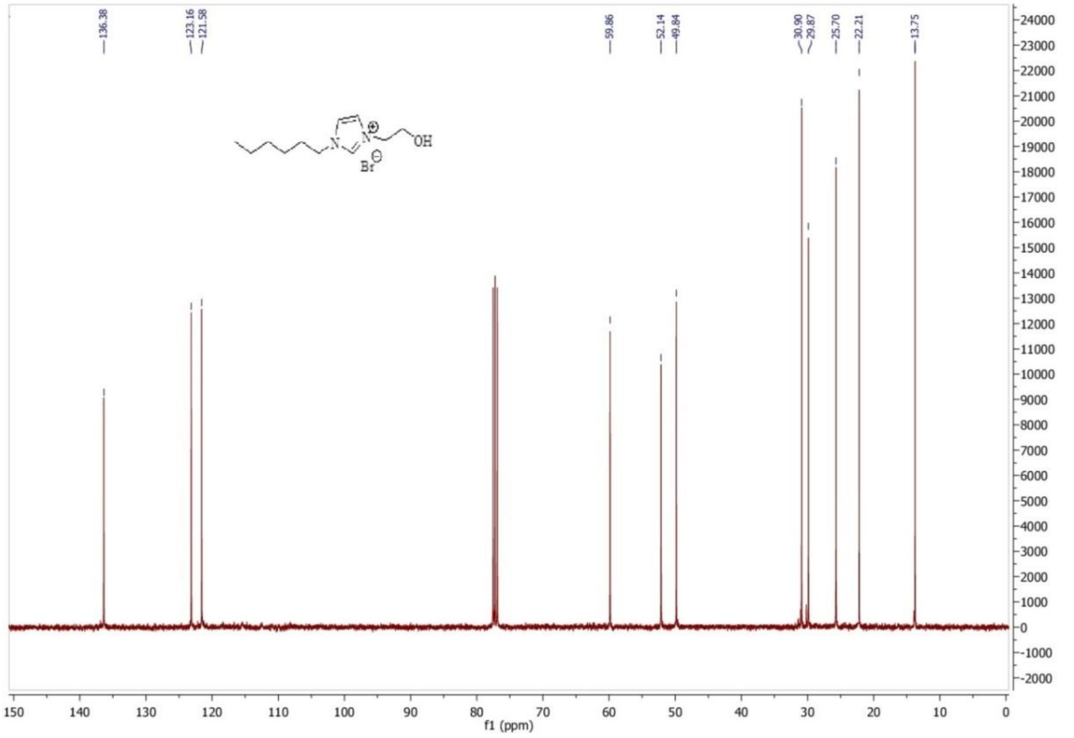

Figure S26. ^{13}C NMR spectrum of IL **7** in $CDCl_3$ (100 MHz)

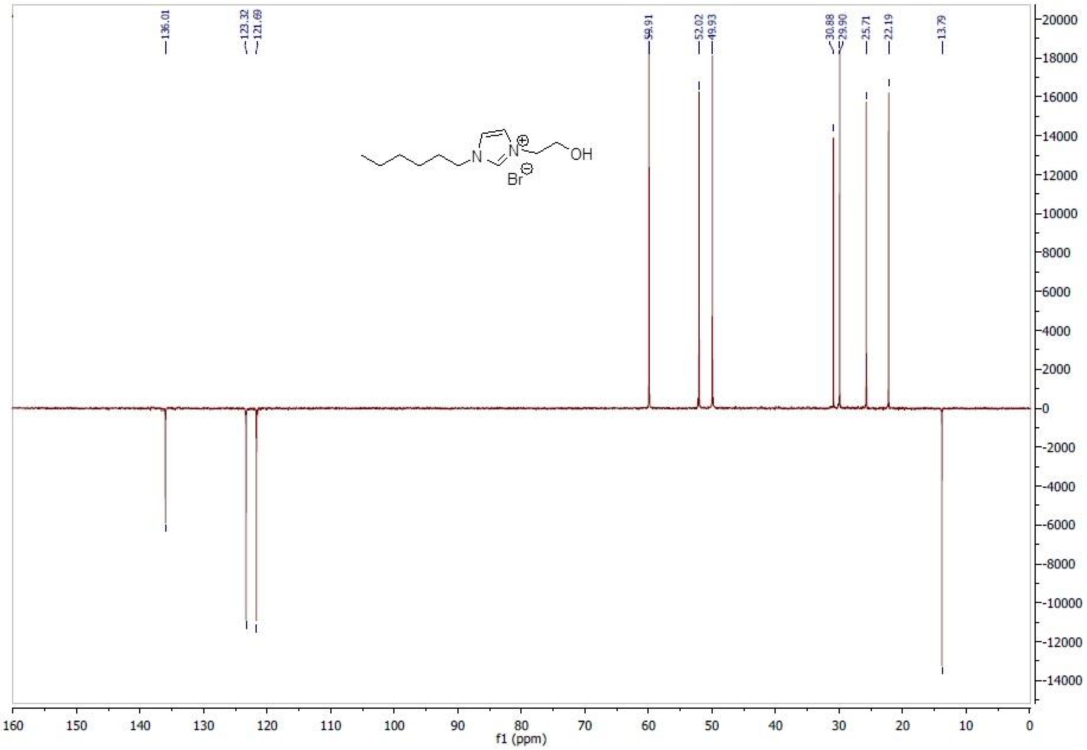

Figure S27. ^{13}C-APT- NMR spectrum of IL **7** in $CDCl_3$ (100 MHz)

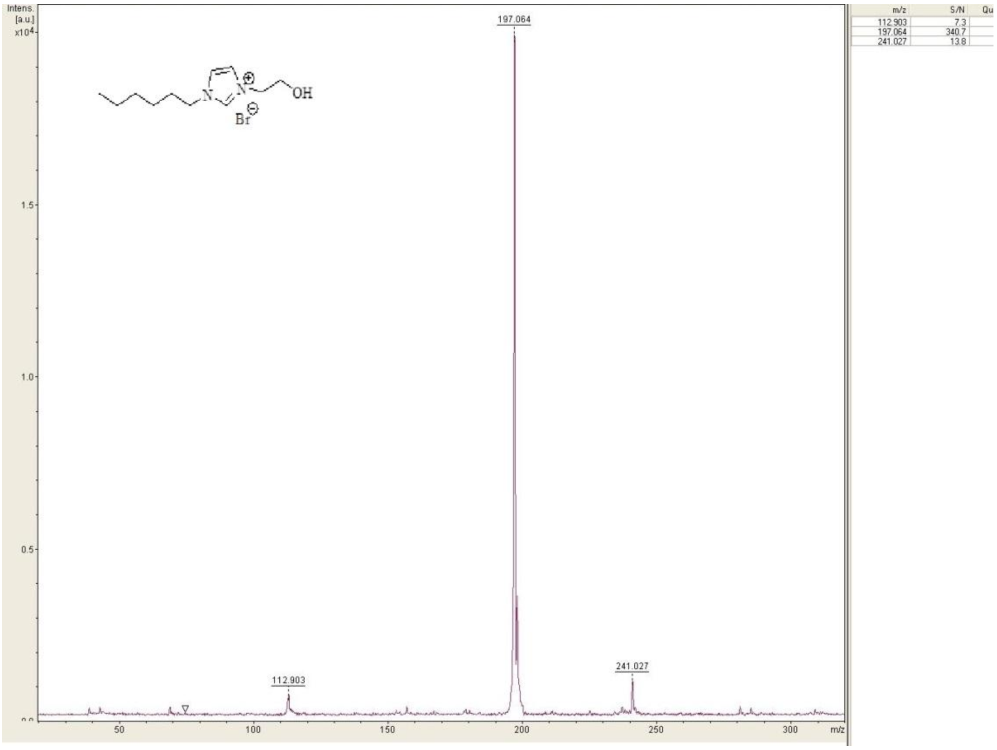

Figure S28. Mass spectrum of IL **7**

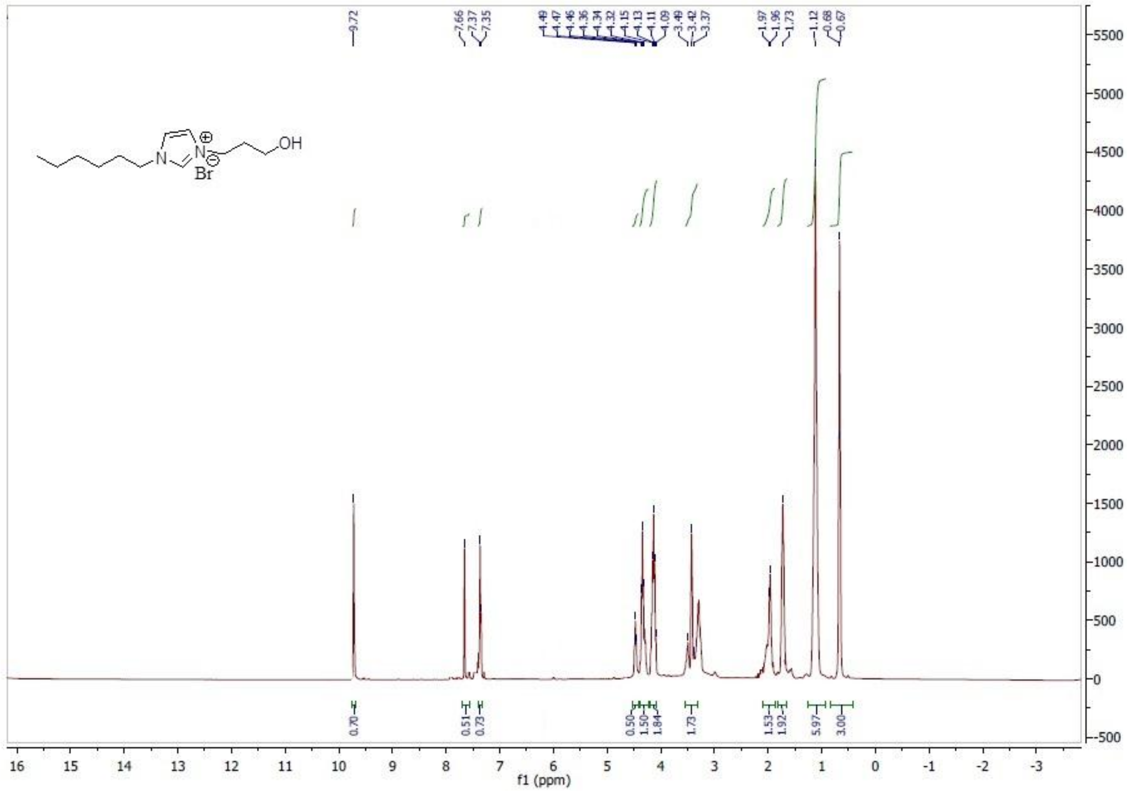

Figure S29. ^{1}H NMR spectrum of IL **8** in $CDCl_3$ (400 MHz)

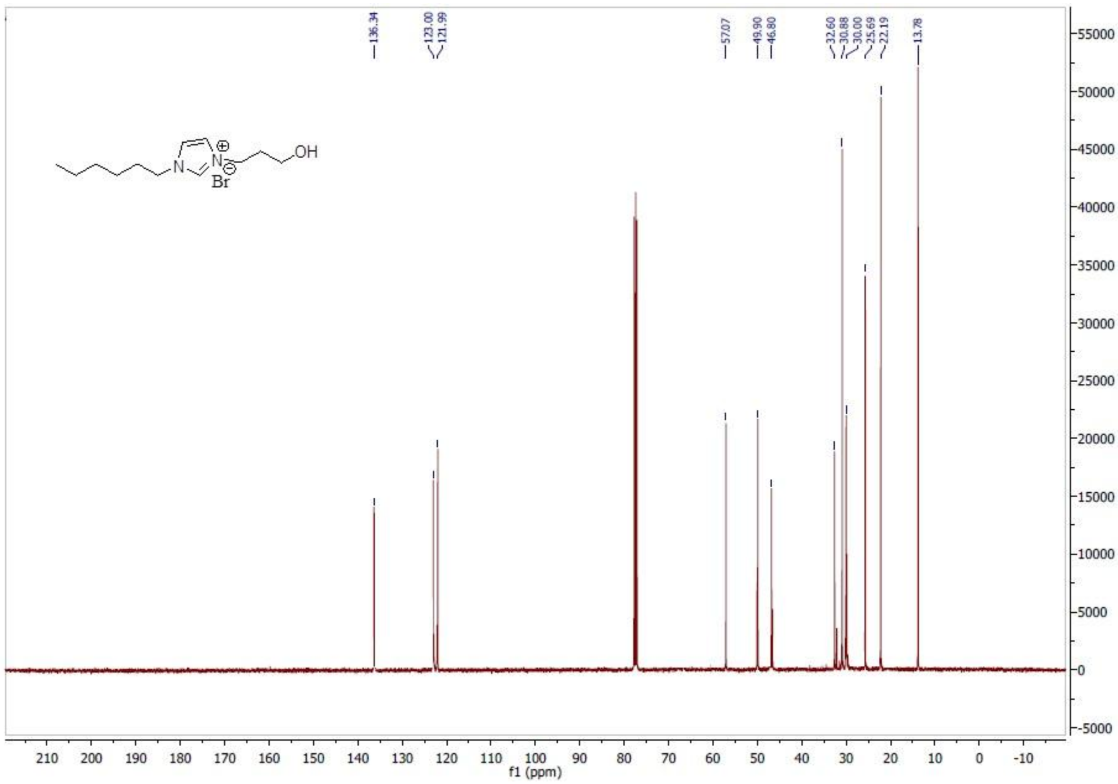

Figure S30. ^{13}C NMR spectrum of IL **8** in $CDCl_3$ (100 MHz)

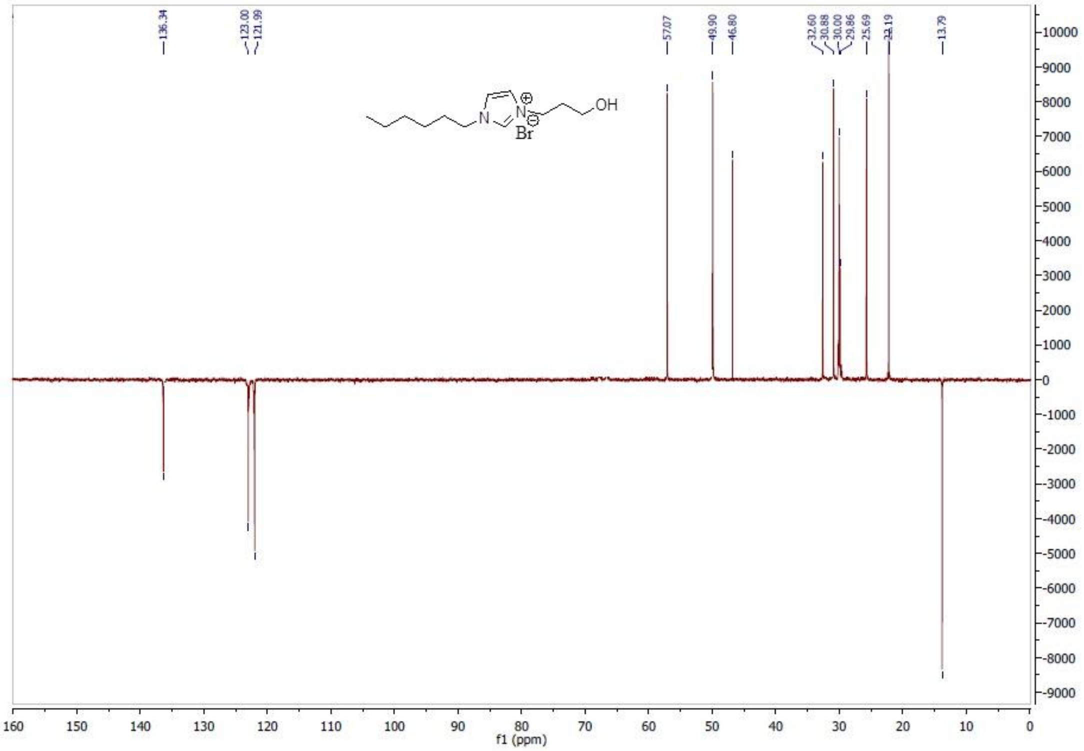

Figure S31. ^{13}C-APT- NMR spectrum of IL **8** in $CDCl_3$ (100 MHz)

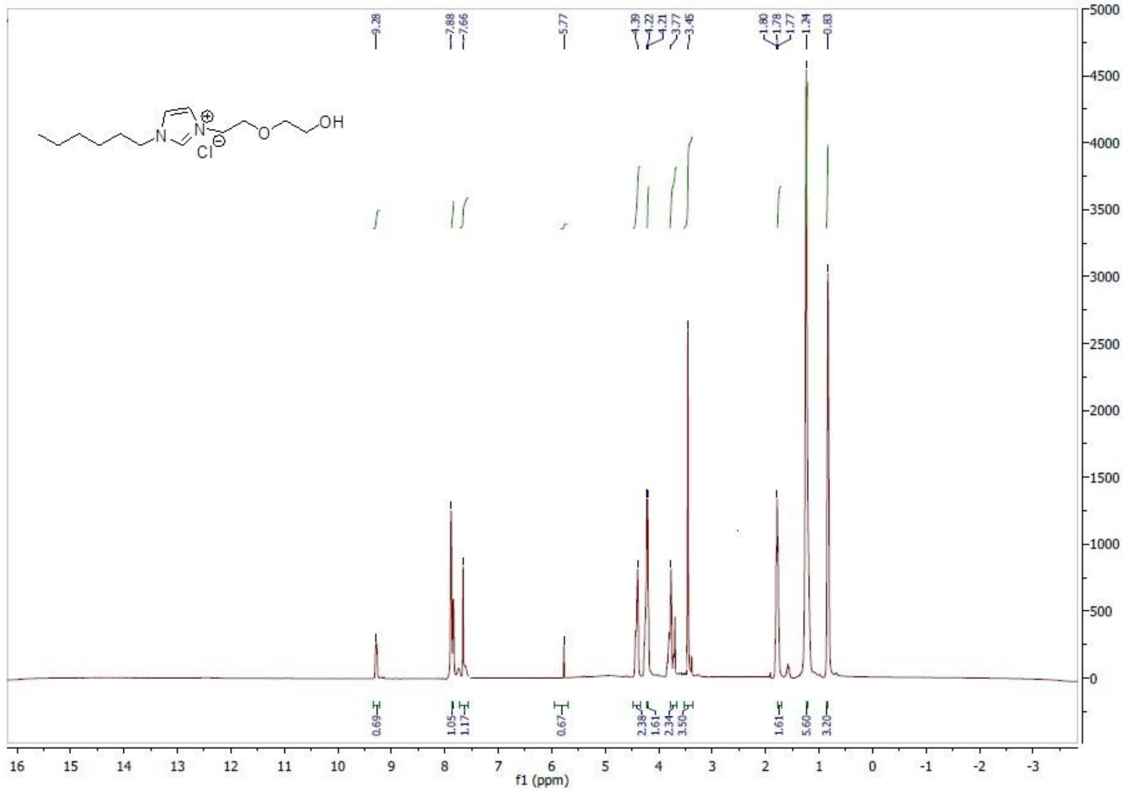

Figure S32. ^{1}H NMR spectrum of IL **9** in DMSO-d_6 (400 MHz)

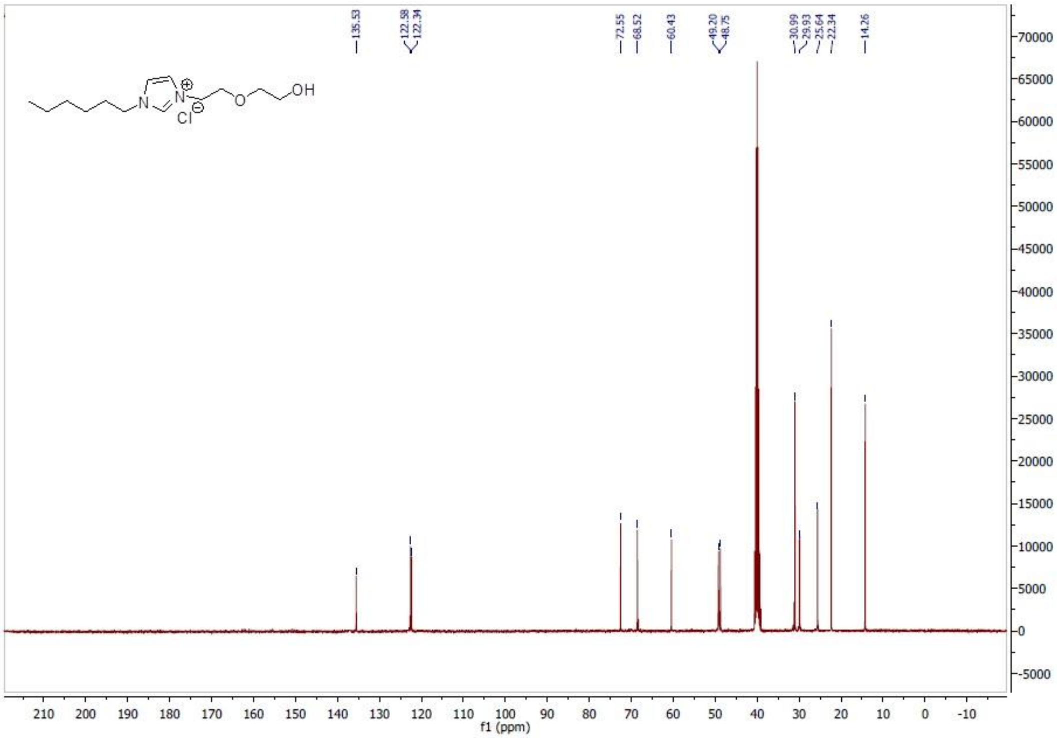

Figure S33. ^{13}C NMR spectrum of IL **9** in DMSO-d_6 (100 MHz)

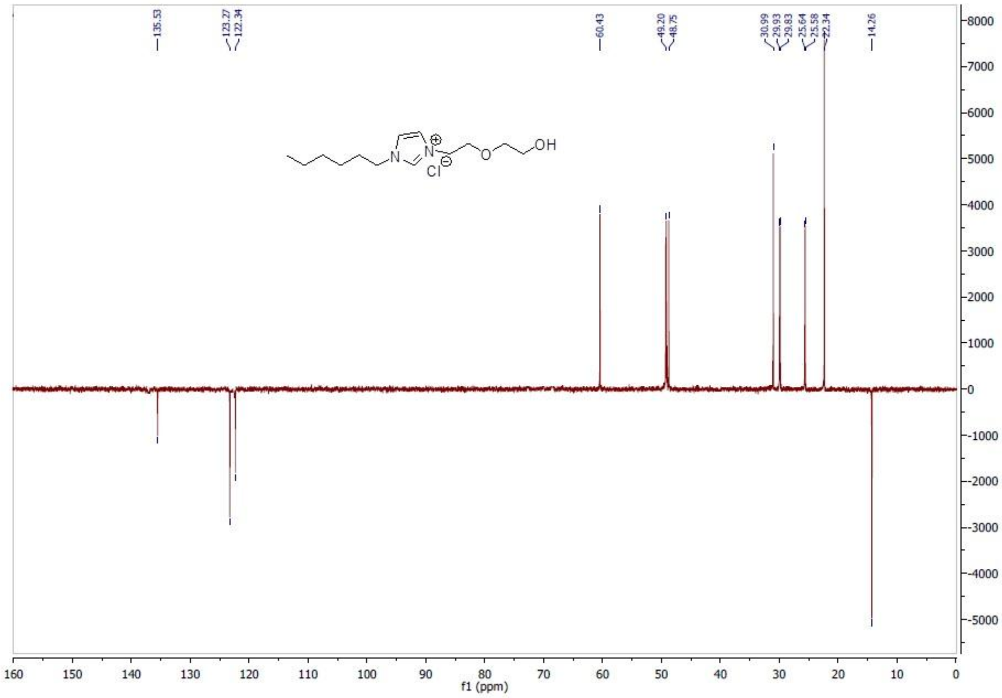

Figure S34. ^{13}C-APT- NMR spectrum of IL **9** in DMSO-d_6 (100 MHz)

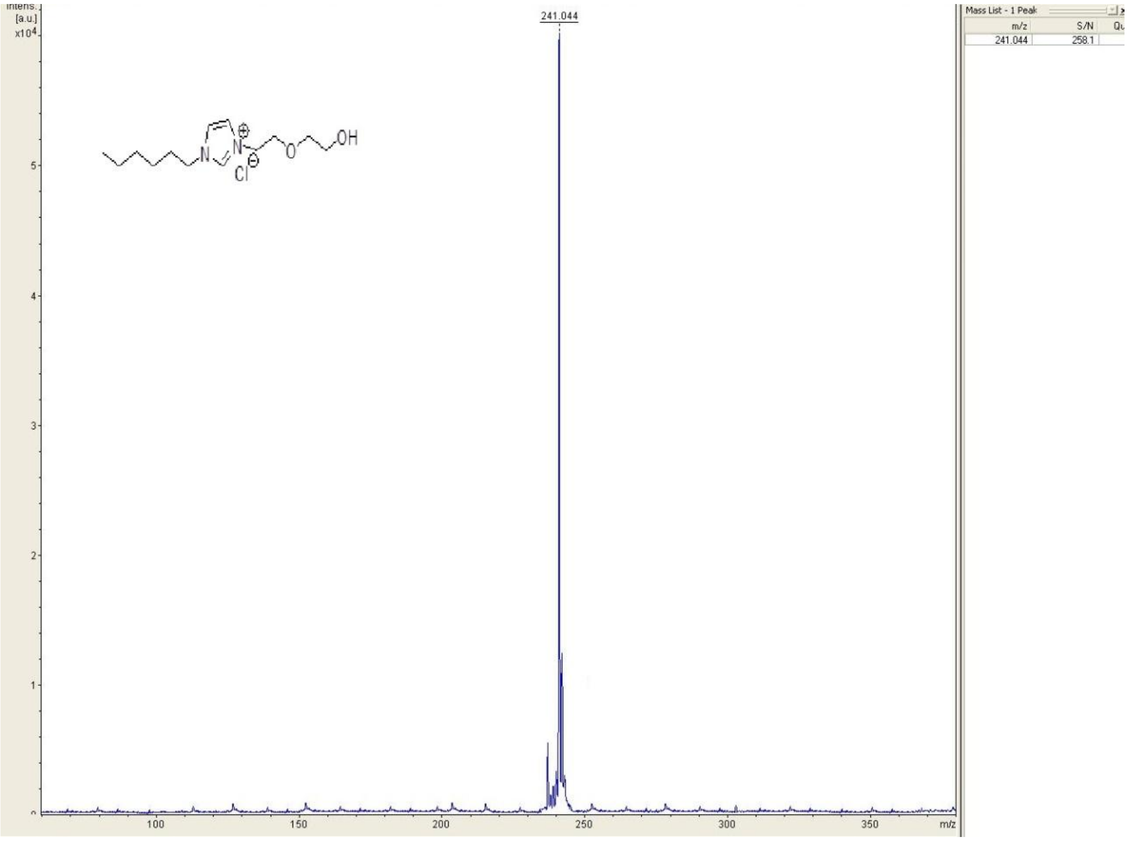

Figure S35. Mass spectrum of IL **9**

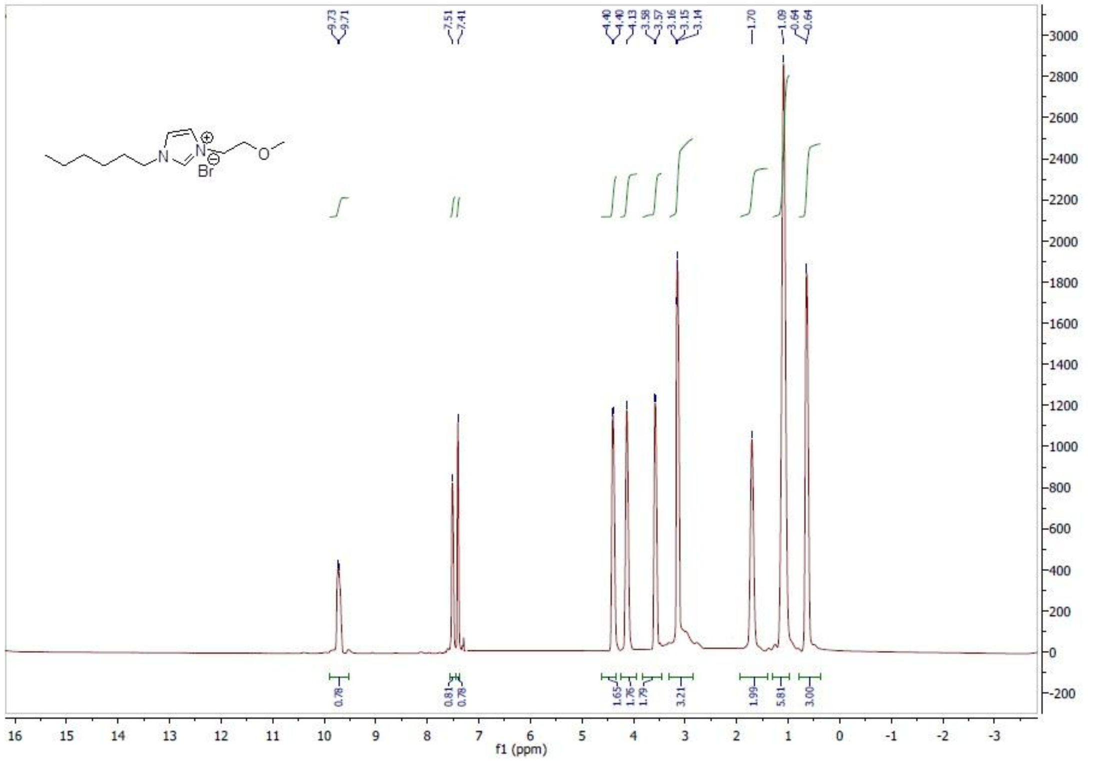

Figure S36. ^{1}H NMR spectrum of IL **10** in $CDCl_3$ (400 MHz)

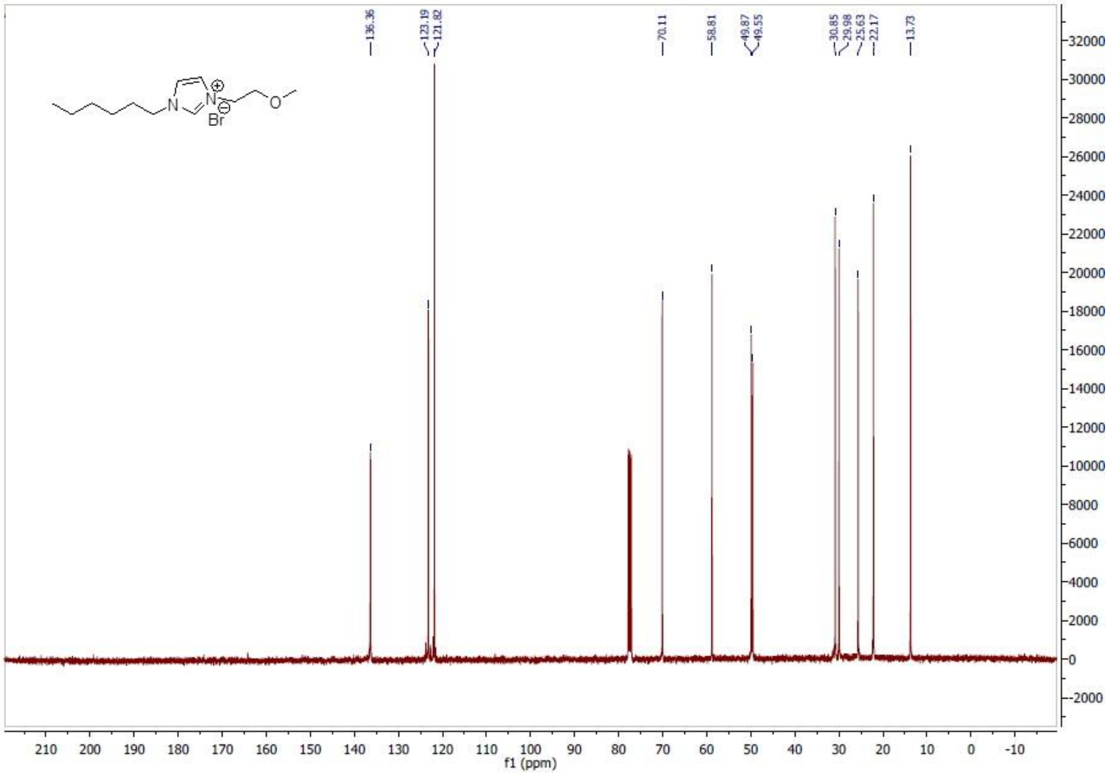

Figure S37. ^{13}C NMR spectrum of IL **10** in $CDCl_3$ (100 MHz)

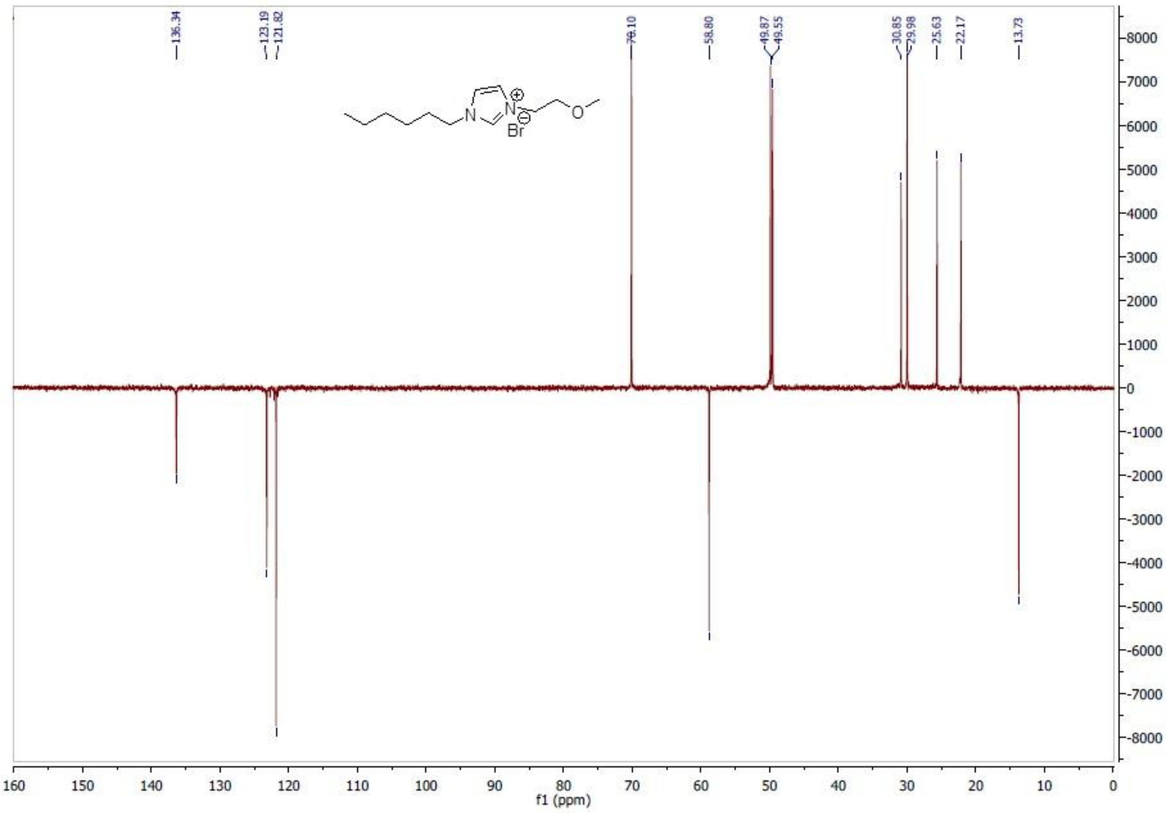

Figure S38. ^{13}C-APT- NMR spectrum of IL **10** in $CDCl_3$ (100 MHz)

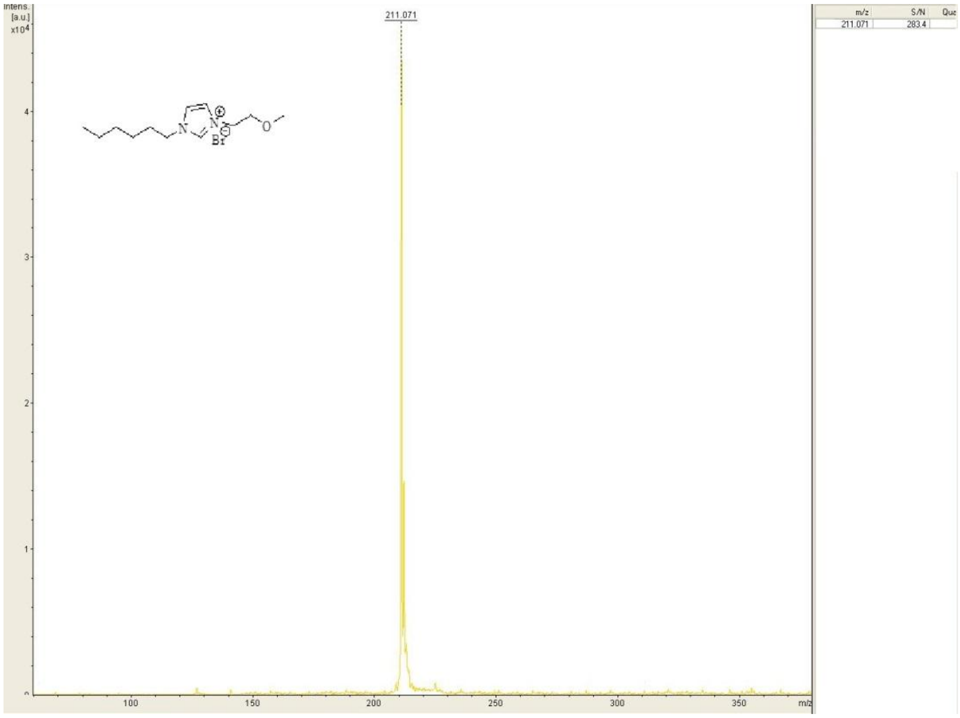

Figure S39. Mass spectrum of IL **10**

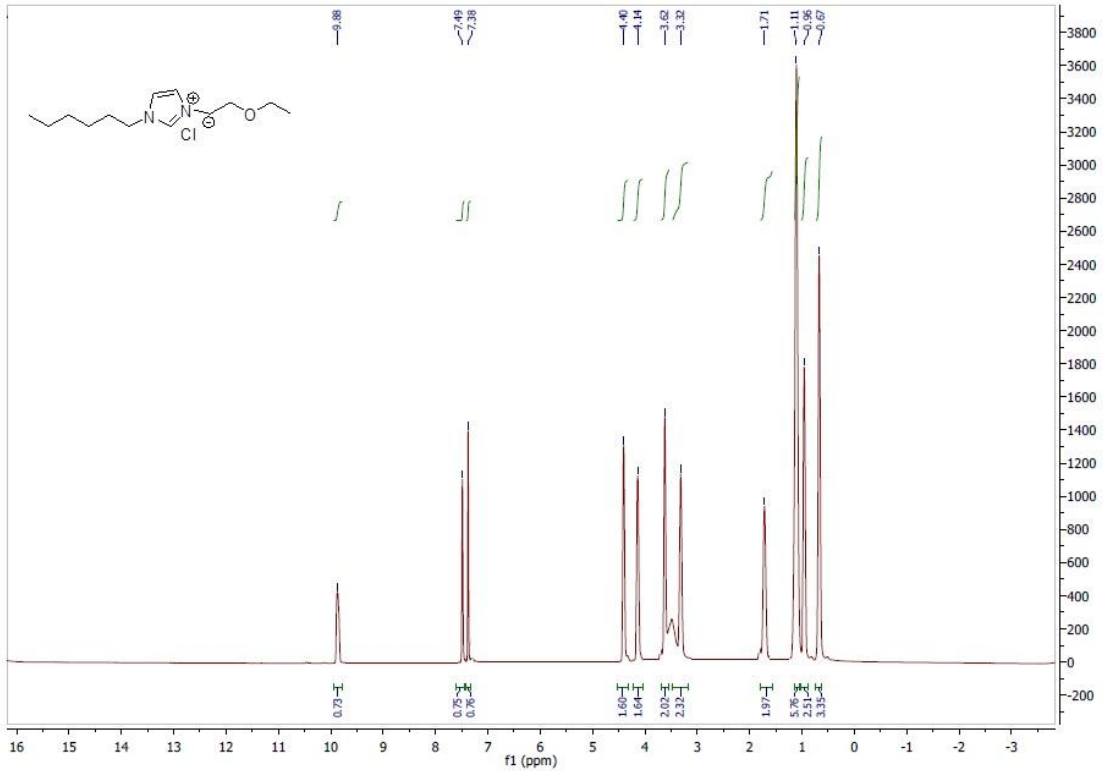

Figure S40. ^{1}H NMR spectrum of IL **11** in $CDCl_3$ (400 MHz)

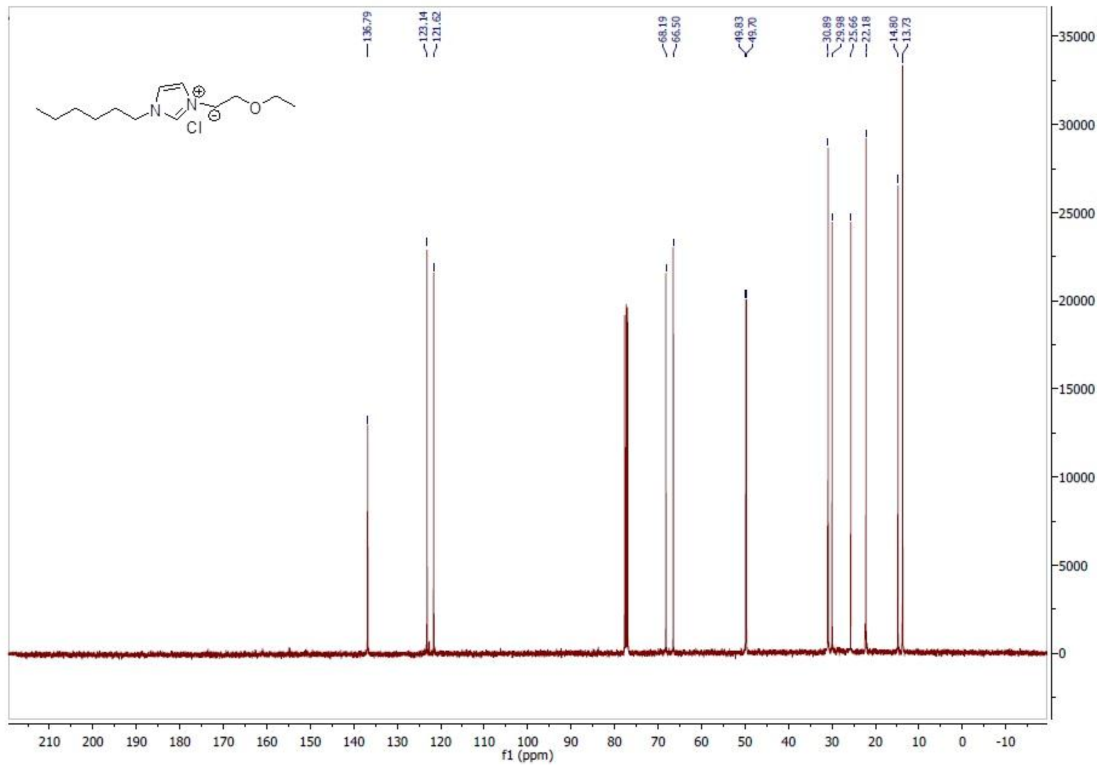

Figure S41. ^{13}C NMR spectrum of IL **11** in $CDCl_3$ (100 MHz)

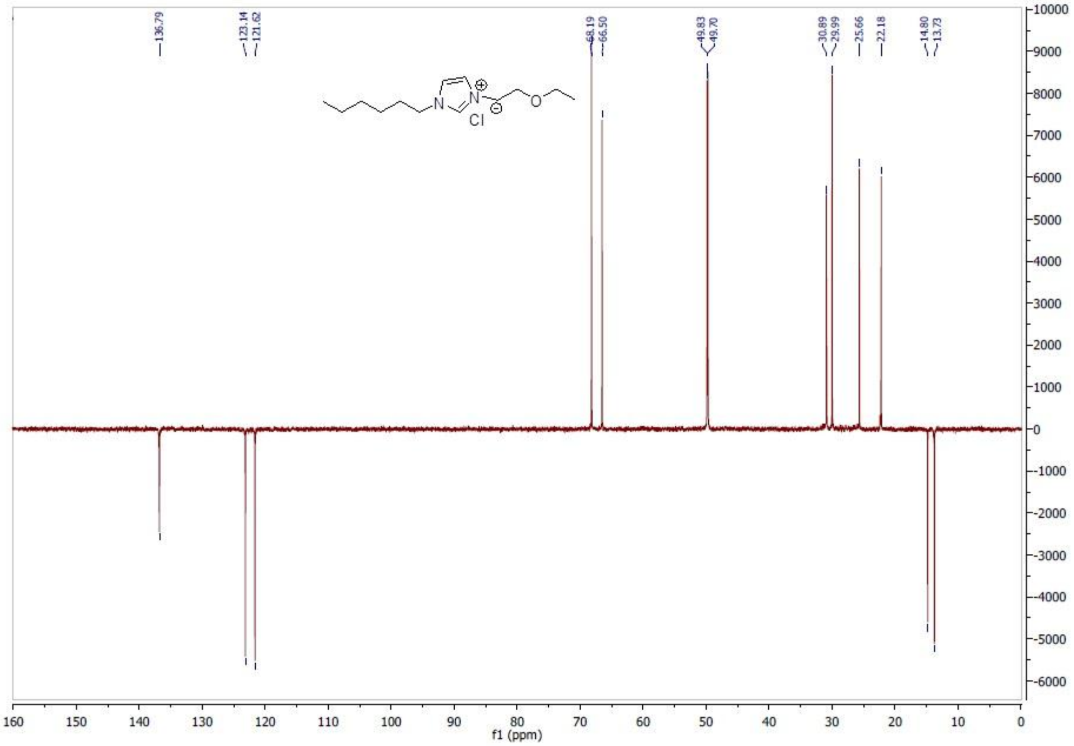

Figure S42. ^{13}C-APT- NMR spectrum of IL **11** in $CDCl_3$ (100 MHz)

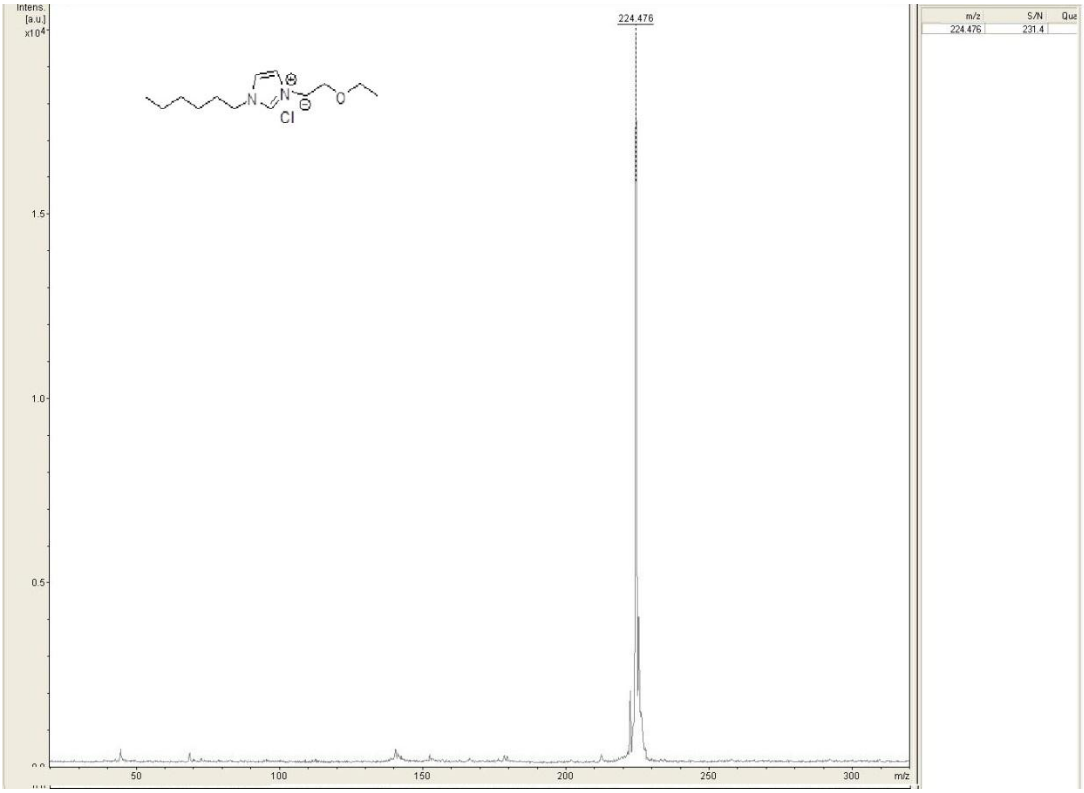

Figure S43. Mass spectrum of IL **11**

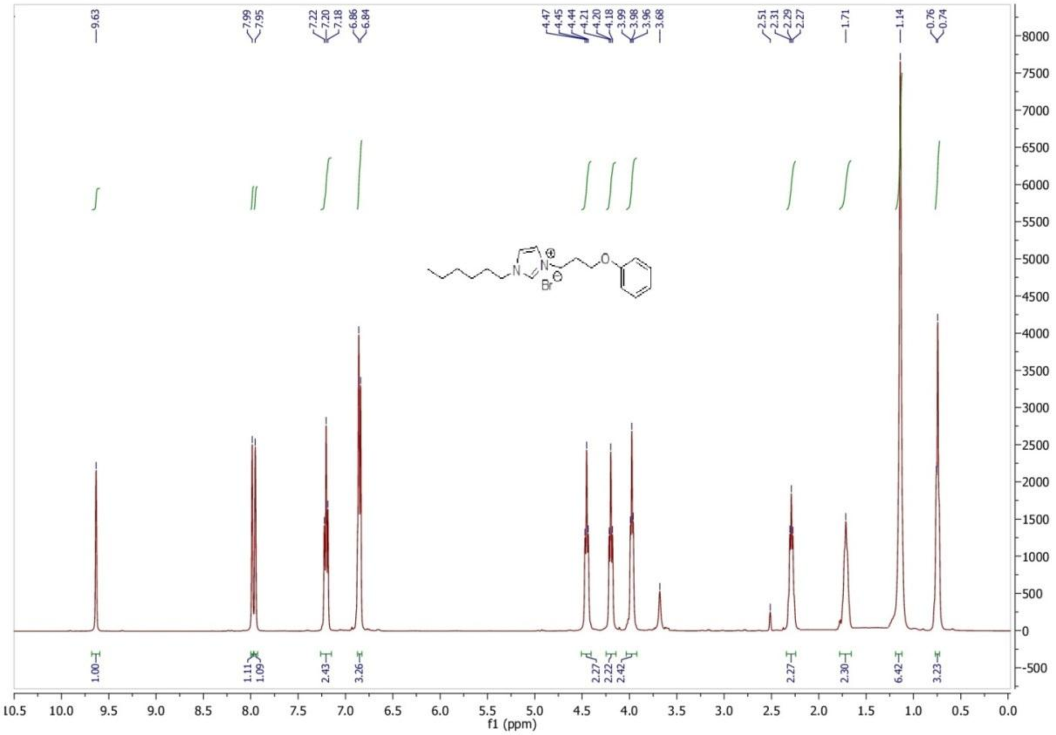

Figure S44. ^{1}H NMR spectrum of IL **12** in DMSO-d_6 (400 MHz)

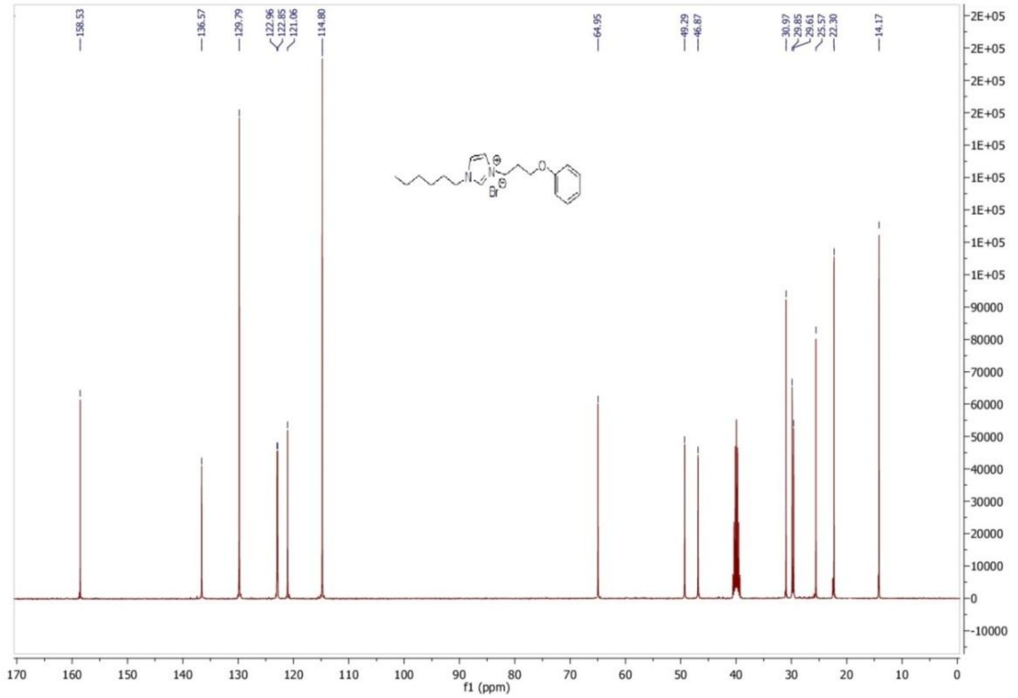

Figure S45. ^{13}C NMR spectrum of IL **12** in DMSO-d_6 (100 MHz)

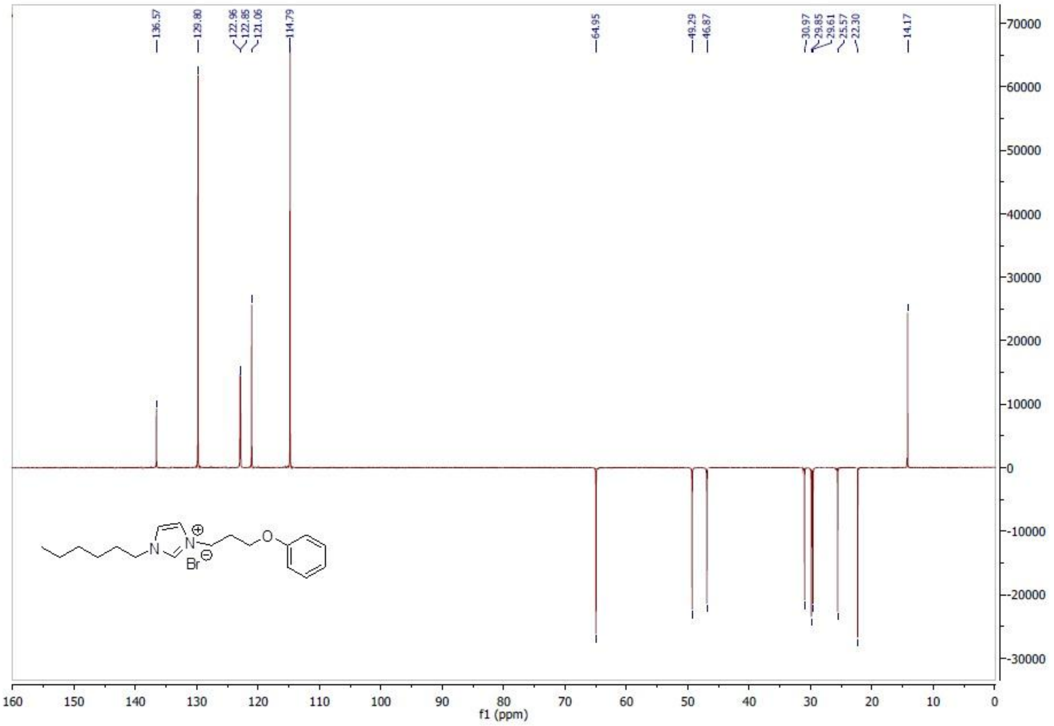

Figure S46. ^{13}C-DEPT- NMR spectrum of IL **12** in DMSO-d_6 (100 MHz)

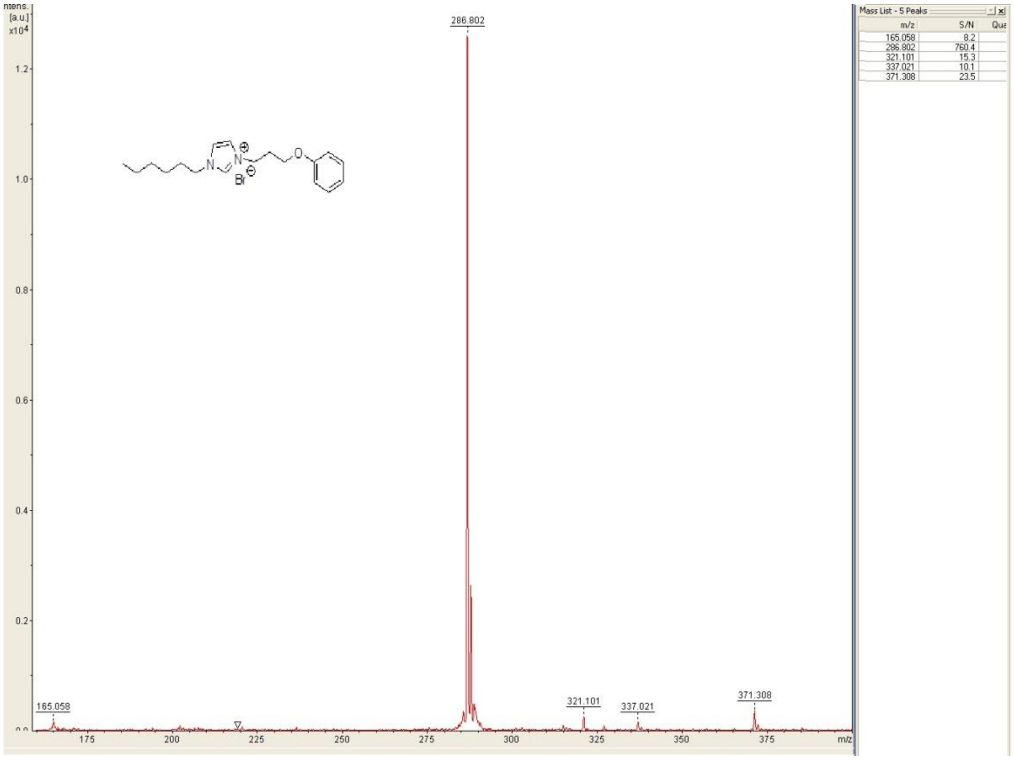

Figure S47. Mass spectrum of IL **12**

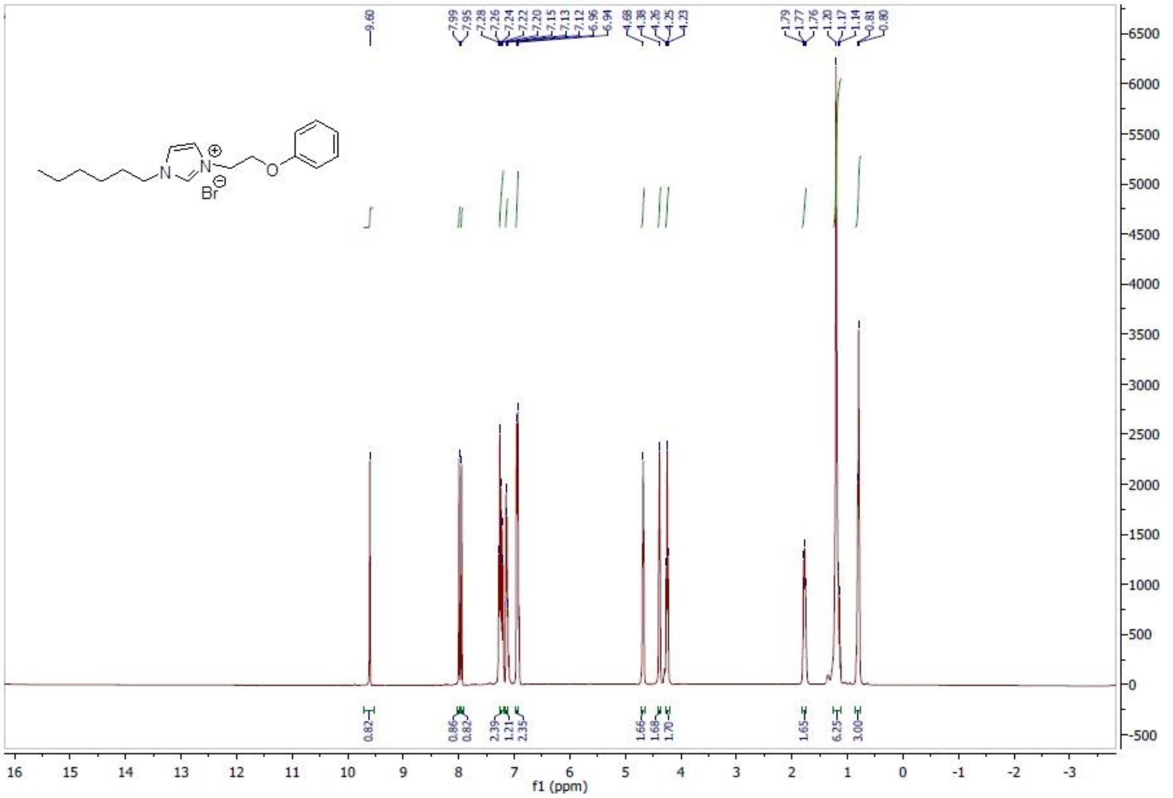

Figure S48. ^{1}H NMR spectrum of IL **13** in DMSO-d_6 (400 MHz)

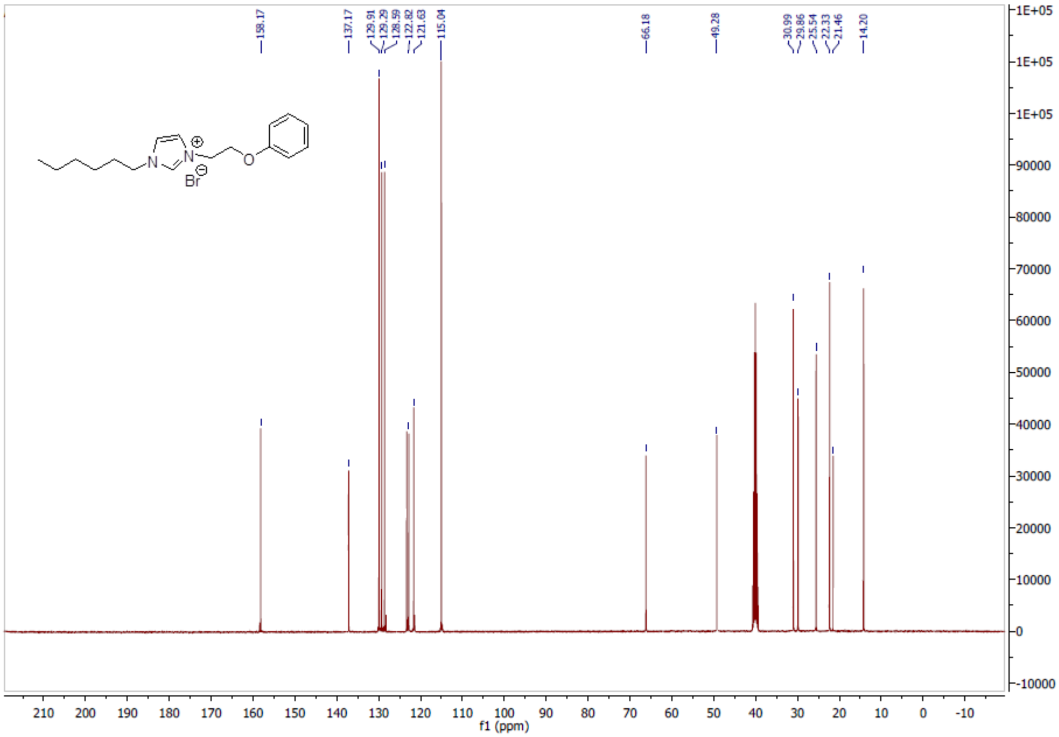

Figure S49. ^{13}C NMR spectrum of IL **13** in DMSO-d_6 (100 MHz)

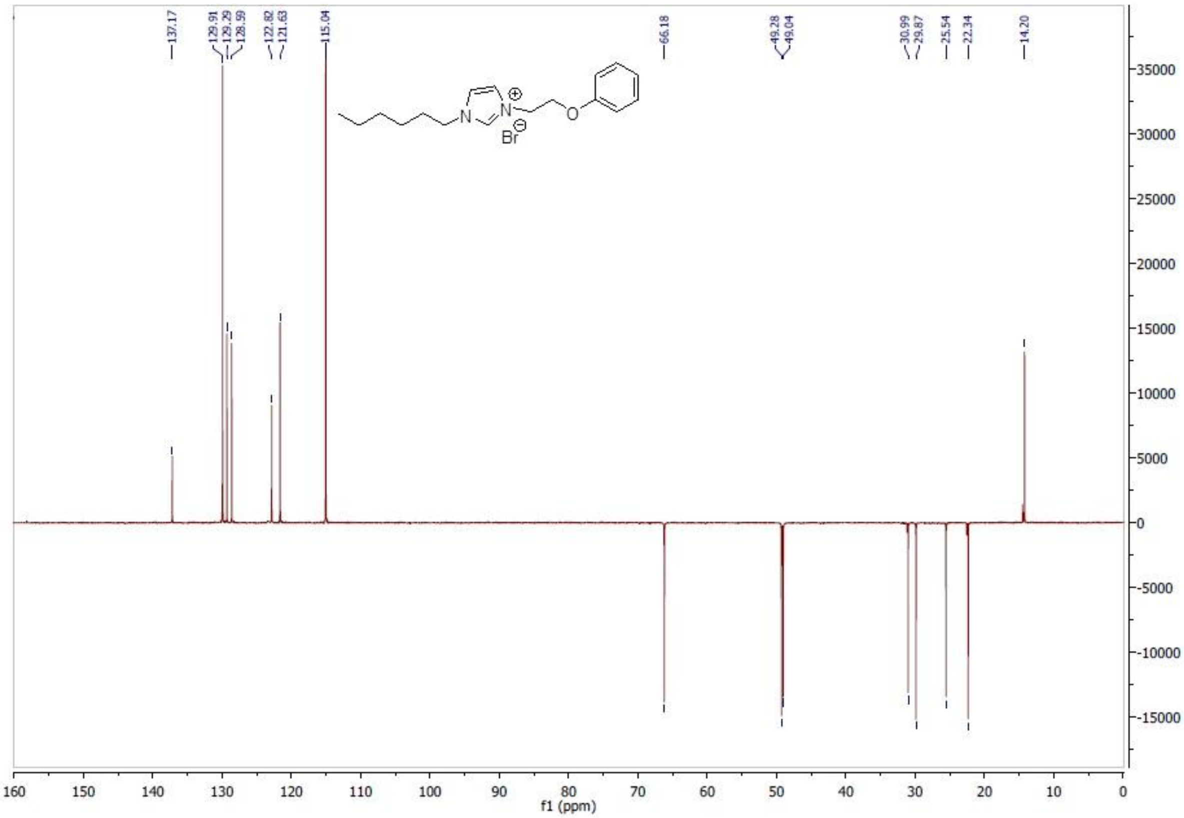

Figure S50. ^{13}C-DEPT- NMR spectrum of IL **13** in DMSO-d_6 (100 MHz)

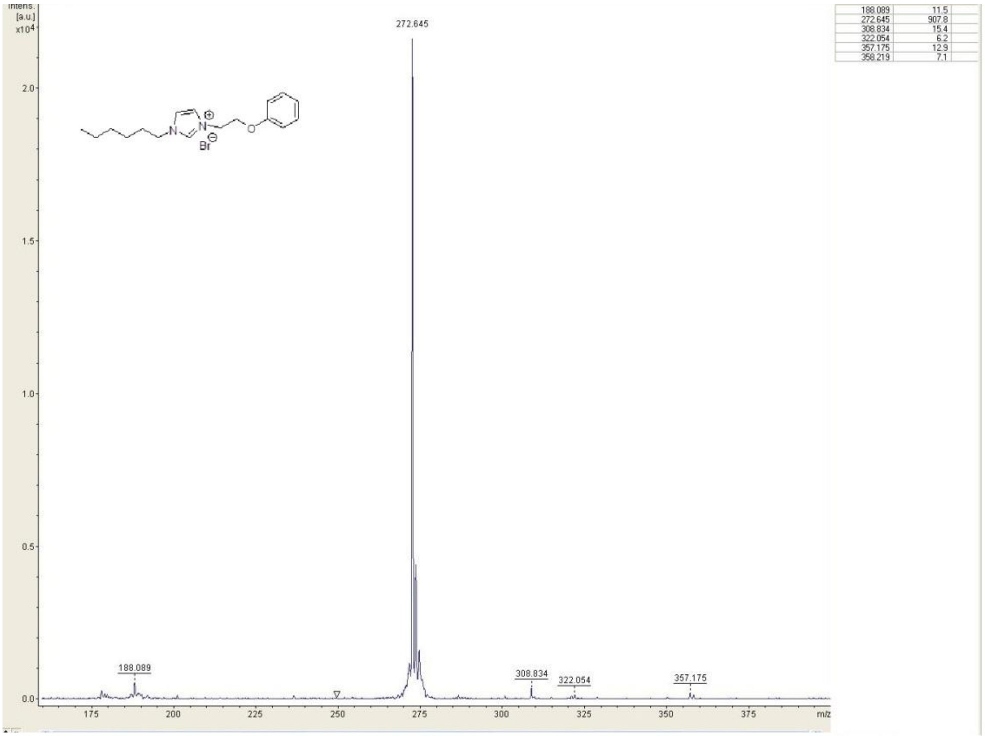

Figure S51. Mass spectrum of IL **13**

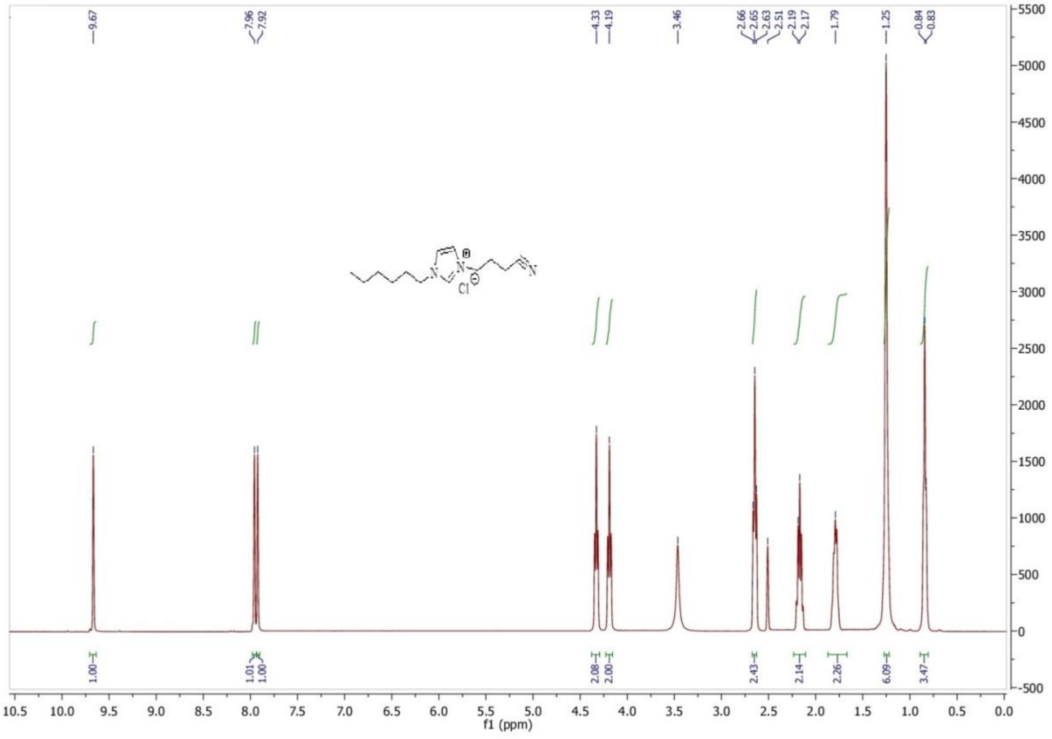

Figure S52. ^{1}H NMR spectrum of IL **14** in DMSO-d_6 (400 MHz)

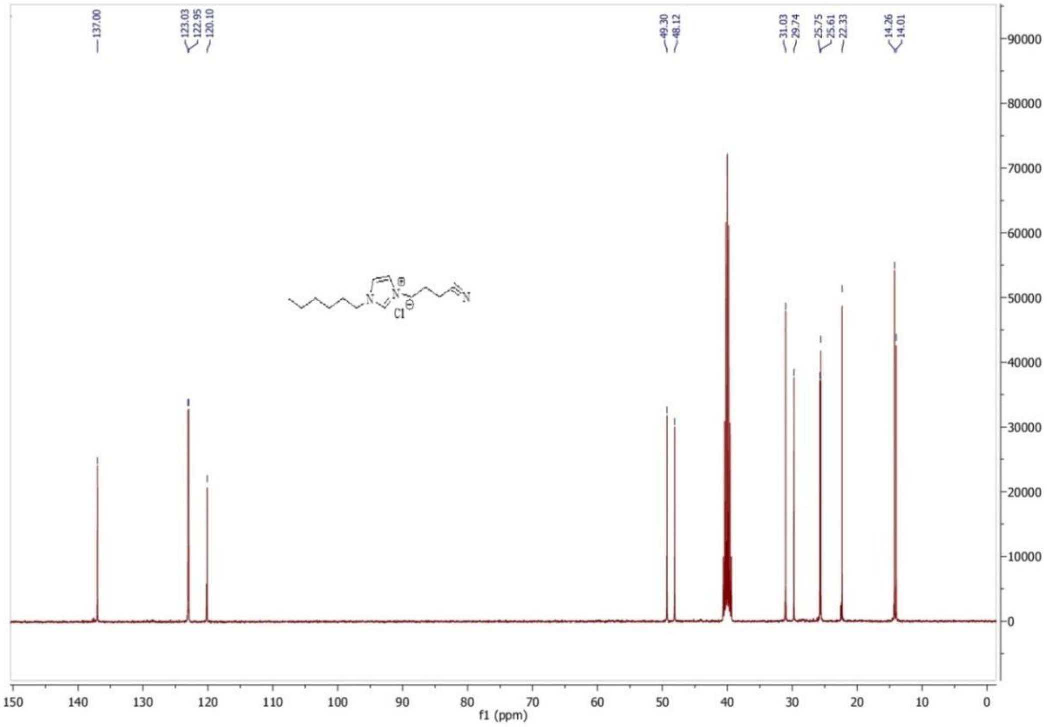

Figure S53. ^{13}C NMR spectrum of IL **14** in DMSO-d_6 (100 MHz)

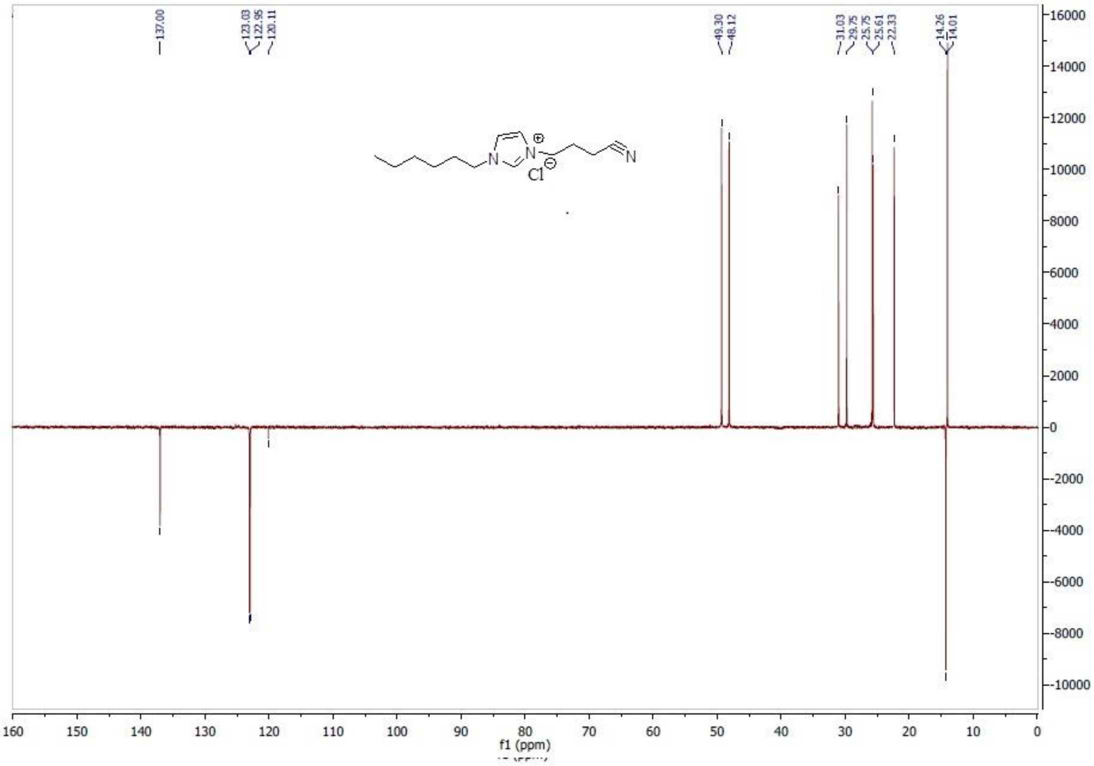

Figure S54. ^{13}C-APT- NMR spectrum of IL **14** in DMSO-d_6 (100 MHz)

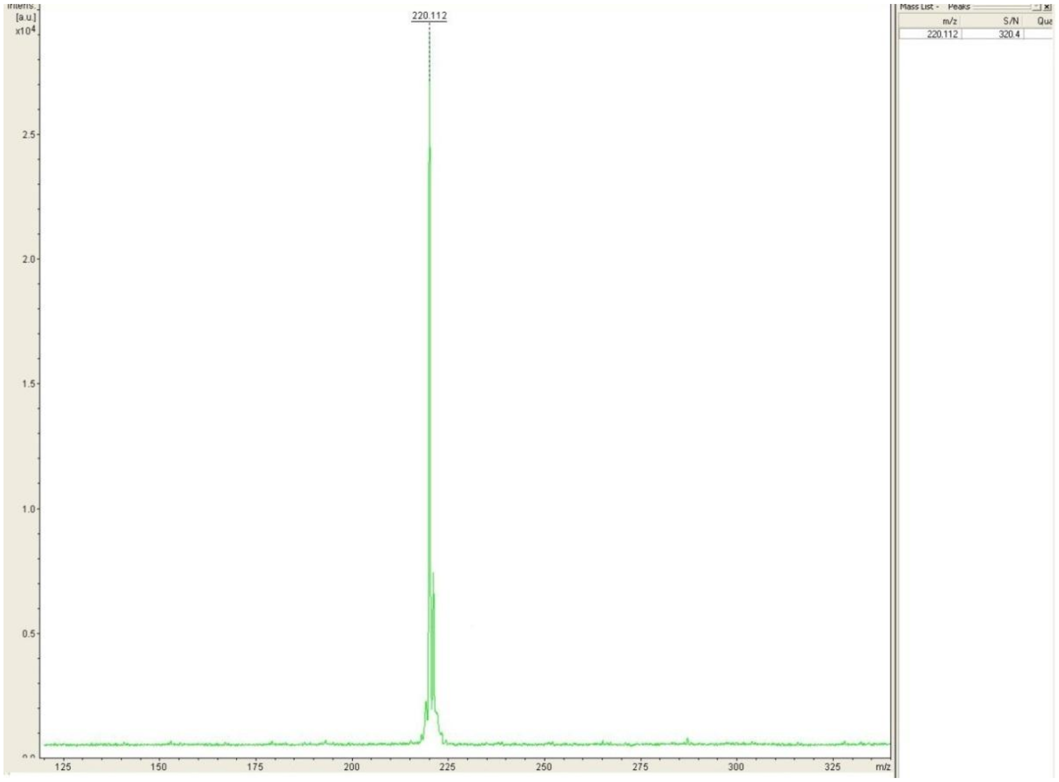

Figure S55. Mass spectrum of IL **14**
